# Haploidentical vs. HLA-matched donor hematopoietic stem-cell transplantation for pediatric patients with acute lymphoblastic leukemia in second remission: A collaborative retrospective study of the Spanish Group for Bone Marrow Transplantation in Children (GETMON/GETH) and the Spanish Childhood Relapsed ALL Board (ReALLNet)

**DOI:** 10.3389/fped.2023.1140637

**Published:** 2023-03-20

**Authors:** Celia Moreno, Eduardo Ramos-Elbal, Pablo Velasco, Yurena Aguilar, Berta Gonzáález Martínez, Carolina Fuentes, Águeda Molinos, Pilar Guerra-García, Pilar Palomo, Jaime Verdu, Rosa María Adán Pedroso, José Manuel Vagace, Mónica López-Duarte, Alexandra Regueiro, María Tasso, José Luis Dapena, José Antonio Salinas, Samuel Navarro, Francisco Bautista, Álvaro Lassaletta, Francisco Lendínez, Susana Rives, Antonia Pascual, Antonia Rodríguez, José María Pérez-Hurtado, José María Fernández, Antonio Pérez-Martínez, Marta González-Vicent, Cristina Díaz de Heredia, José Luis Fuster

**Affiliations:** ^1^ Hospital Clínico Universitario Virgen de la Arrixaca, Murcia, Spain; ^2^ Hospital Universitario Vall d’Hebron, Vall d’Hebron Institut de Recerca, Barcelona, Spain; ^3^ Hospital Universitario Miguel Servet, Zaragoza, Spain; ^4^ Hospital Universitario La Paz, IdiPAZ, Instituto de Investigación, Hospital Universitario La Paz, Madrid, Spain; ^5^ Hospital Universitario y Politécnico La Fe, Valencia, Spain; ^6^ Hospital Universitario Virgen del Rocío, Sevilla, Spain; ^7^ Hospital Universitario 12 de octubre, Madrid, Spain; ^8^ Hospital Universitario Central de Asturias, Oviedo, Spain; ^9^ Hospital Universitario de Valencia, Valencia, Spain; ^10^ Hospital Universitario de Cruces, Vizcaya, Spain; ^11^ Complejo Hospitalario Universitario de Badajoz, Badajoz, Spain; ^12^ Hospital de Valdecilla, Instituto de Investigación Sanitaria Valdecilla, IDIVAL, Santander, Spain; ^13^ Hospital Clínico Universitario de Santiago, Santiago de Compostela, Spain; ^14^ Hospital General Universitario Doctor Balmis, Alicante, Spain; ^15^ Pediatric Cancer Center, Hospital Sant Joan de Déu, Barcelona, Spain; ^16^ Institut de Recerca San Joan de Déu, Barcelona, Spain; ^17^ Hospital Universitario Son Espases, Palma de Mallorca, Spain; ^18^ Prinses Maxima Center, Utrech, Netherlands; ^19^ Hospital Universitario Niño Jesús, Madrid, Spain; ^20^ Hospital Universitario Torrecárdenas, Almería, Spain; ^21^ Hospital Regional Universitario de Málaga, Málaga, Spain; ^22^ Hospital Universitario Reina Sofía, Córdoba, Spain; ^23^ Instituto Murciano de Investigación Biosanitaria (IMIB), Murcia, Spain

**Keywords:** acute lymphoblastic leukemia, relapse, children, stem cell transplantation, haploidentical, donor

## Abstract

**Introduction:**

Studies addressing the role of haploidentical as alternative to HLA-matched donors for stem cell transplantation (SCT) often include patients with diverse hematological malignancies in different remission statuses.

**Methods:**

We compared outcomes of children with acute lymphoblastic leukemia (ALL) undergoing SCT in second complete remission (CR2) from haploidentical (n = 25) versus HLA-matched donor (n = 51).

**Results:**

Patients were equally distributed across both groups according to age, immunophenotype, time to and site of relapse, relapse risk-group allocation, and minimal residual disease (MRD) before SCT. Incidence of graft failure, acute graft versus host disease (GVHD), and other early complications did not differ between both groups. We found no differences in overall survival (58.7% versus 59.5%; *p* = .8), leukemia free survival (LFS) (48% versus 36.4%; *p* = .5), event free survival (40% versus 34.4%; *p* = .69), cumulative incidence (CI) of subsequent relapse (28% versus 40.9%; *p* = .69), treatment related mortality (24% versus 23.6%; *p* = .83), CI of cGVHD (4.5% versus 18.7%; *p* = .2), and chronic GVHD-free and leukemia-free survival (44% versus 26.3%; *p* = .3) after haploidentical donor SCT. Chronic GVHD (HR = 0.09; *p*=.02) had protective impact, and MRD ≥ 0.01% before SCT (HR = 2.59; *p*=.01) had unfavorable impact on LFS.

**Discussion:**

These results support the role of haploidentical donor SCT in children with ALL in CR2.

## Introduction

Allogeneic stem cell transplantation (SCT) is well established as a consolidation treatment for children with high-risk (HR) and refractory/relapsed acute lymphoblastic leukemia (ALL) (1, 2).

Historically, in order to prevent graft rejection and graft vs. host disease (GVHD), human leukocyte antigen (HLA)-matched related or unrelated donors were preferred for SCT (1, 3–7).

Haploidentical donors represent an alternate option for those patients lacking a suitable and timely ready-matched donor, a critical aspect in children after relapse of ALL (4–7).

Both post-transplant cyclophosphamide administration after T-cell replete infusion and *ex vivo* graft manipulation through different T-cell depletion procedures such as CD3-, αβ T-cell-, and CD45RA-depletion are widely performed for prevention of graft failure and GVHD after haploidentical transplantation in children (8–21). T-cell depletion allows the administration of a large amount of alloreactive natural killer (NK) cells within the graft; moreover, modern *ex vivo* graft manipulation strategies, such as αβ- and CD45RA+ T-cell depletion, yield cell products with γδ- and memory T-cell content, which might help reduce the risk of relapse and infectious complications after SCT (13, 19–22).

Previous reports have addressed the role of haploidentical donor SCT in children. However, most of them included patients with diverse hematological malignancies as well as a substantial number of patients undergoing transplantation in other than second remission, including first remission (8, 10–14, 16–18, 21–26).

A retrospective study found no differences in the treatment outcomes of children with acute leukemia undergoing matched sibling donor SCT and T-cell replete haploidentical grafts with intensified immunological suppression without post-transplant cyclophosphamide (the “Beijing protocol”) (26).

Similarly, Mo et al. did not find differences in outcomes in children with ALL undergoing unmanipulated SCT from haploidentical donors and umbilical cord blood (23).

In contrast, Yanir et al. reported inferior outcomes of pediatric patients with ALL undergoing transplantation after CD34+ selected grafts from haploidentical donors when compared with those transplanted from matched siblings or unrelated donors (2).

In a recent report from the Berlin–Frankfurt–Muenster (BFM) Study Group, children with a very high risk of relapse ALL undergoing SCT from mismatched donors, defined as those with ≥2 allelic or antigenic disparities, including haploidentical donors and <5/6 matches umbilical cord blood units, had worse overall survival (OS), event-free survival (EFS), and nonrelapse mortality than those transplanted from HLA-matched donors (27).

All the aforementioned reports included patients in different remission statuses before SCT.

In the present study, we analyze and compare the treatment outcomes of two cohorts of children with first relapse of ALL included in the Spanish “SEHOP/PETHEMA 2015” registry undergoing transplantation in second complete remission (CR2) from haploidentical vs. HLA-matched donors.

## Patients and methods

### Patients

This is a multicenter retrospective study of children with relapsed ALL included in the “SEHOP/PETHEMA 2015” registry. The main objective was to analyze and compare the outcome of patients undergoing SCT from haploidentical instead of any other related or unrelated HLA-matched donors, including umbilical cord blood.

“SEHOP/PETHEMA 2015” is the Spanish Recommendations Guideline and Registry for children with first relapse of ALL. It was developed in 2015 by the Leukemia Working Group of the Spanish Society of Pediatric Hematology and Oncology (SEHOP) and was approved by the Ethics Committee of the Hospital Clínico Universitario Virgen de la Arrixaca (Murcia, Spain). All patients or their legal guardians provided written informed consent before registration and treatment, in accordance with the Declaration of Helsinki.

These guidelines were based on both “IntReALL SR 2010” (EudraCT Number 2012–000793-30) and “IntReALL HR 2010” (EudraCT Number 2012-000810-12) clinical trials and adopted all diagnostic and response criteria as well as the elements of their standard (noninvestigational) treatment arms.

### Definitions and procedures

The diagnosis and definition of relapse were based on standard criteria (Supplementary Table S1). Relapse after SCT and *BCR-ABL1*-positive ALL were exclusion criteria.

The risk stratification of patients is shown in Table 1. In brief, all patients with very early relapse (<6 months after the end of first-line treatment and <18 months after primary diagnosis), all patients with T-cell immunophenotype and bone marrow (BM) involvement at relapse, and patients with early (<6 months after the end of first-line treatment but ≥18 months after primary diagnosis) isolated BM relapse of B-cell precursor (BCP) immunophenotype were classified as HR relapse. All other patients were classified as standard risk (SR) relapse.

**Table 1:** Definition of risk groups at relapse.

Relapse	Immunophenotype: B-cell precursor			Immunophenotype: T		
Site^a^ Time point^b^	Isolated extramedullary	Bone marrow combined	Bone marrow isolated	Isolated extramedullary	Bone marrow combined	Bone marrow isolated
Very early	HR	HR	HR	HR	HR	HR
Early	SR	SR	HR	SR	HR	HR
Late	SR	SR	SR	SR	HR	HR

HR, high risk; SR, standard risk.

^a^ Isolated extramedullary: extramedullary involvement and <5% blasts in bone marrow (M1 bone marrow). Bone marrow combined: extramedullary involvement and ≥5% blasts in bone marrow (M2 if ≥5% and <25% blasts, M3 if ≥25% blasts).

^b^ Very early relapse: <6 months after the end of first-line treatment and <18 months after primary diagnosis. Early relapse: <6 months after the end of first-line treatment but ≥18 months after primary diagnosis. Late relapse: ≥6 months after the end of first-line treatment.

CR2 was defined as the presence of <5% leukemic blasts in the cytological evaluation of a representative BM sample in the absence of extramedullary persistent disease. Minimal residual disease (MRD) response was assessed by using local flow cytometry after each chemotherapy block and before SCT. An MRD < 0.01% (<10^−4^) was classified as “negative” before SCT.

HR patients received “R3” reinduction chemotherapy followed by three cycles of consolidation chemotherapy according to the IntReALL HR 2010 protocol before SCT (Supplementary Table S2) (28). HR relapse patients were allowed to participate in a randomized phase 3 clinical study (NCT02393859) and receive one cycle of blinatumomab instead of the third consolidation block before SCT.

SR patients received the Acute Lymphoblastic Leukemia Relapse BFM 2002 (ALL-REZ BFM 2002) protocol (Supplementary Table S2), and transplantation was indicated after three cycles of consolidation chemotherapy in those patients with marrow involvement at relapse and poor response to reinduction defined as MRD ≥ 0.1% (≥10^−3^) at day 29 and in those with early combined BM relapse without available MRD evaluation at day 29 (29, 30). For patients with early isolated extramedullary relapse, early combined BM and good response to reinduction, and late BM relapse (isolated or combined) without evaluation of MRD response to reinduction, transplantation was recommended only if an HLA-matched donor was available (Supplementary Table S3).

HLA-matched donors (related or unrelated) were defined as those with ≥ 9/10 HLA matching alleles for BM or peripheral blood stem cells and ≥5/6 for umbilical cord blood grafts (2, 31, 32). Although HLA-matched donors were first recommended, haploidentical donors were also accepted as alternate donors in the absence of an HLA-matched donor or according to the preferences of each institution/investigator.

These guidelines provided no recommendations for transplantation procedures and supportive care. For patients with central nervous system and/or testicular involvement at relapse, cranial or craniospinal, and/or testicular irradiation were recommended during conditioning or after SCT (early Orchiectomy was allowed as an alternative to testicular irradiation).

Primary and secondary graft failures were diagnosed in those patients in whom an absolute neutrophil count >500/µL by day 28 was not reached and in those who lost their primary engraftment after day 28, respectively.

The diagnosis and grading of acute and chronic GVHD and sinusoidal obstructive syndrome (SOS) were established by local investigators according to standard criteria (33–38).

Other moderate and severe adverse events after transplantation were graded according to the National Cancer Institute Common Terminology Criteria for Adverse Events (CTCAE) version 3.0.

Second relapse was defined following the same criteria applied for diagnosis of first relapse (Supplementary Table S1).

### Statistics

The two proportions Z and the Fisher exact tests were used to compare the categorical variables of patients undergoing transplantation from haploidentical vs. HLA-matched donors; patients without available data were excluded from the analysis of the corresponding variable.

OS was defined as the probability of survival after transplantation and was calculated considering the date of transplant and the date of death from any cause or last contact; leukemia-free survival (LFS) was calculated considering the date of transplant and subsequent relapse or death from any cause as events; graft failure and second malignant neoplasm were included as additional events for the calculation of EFS. Patients lost to follow-up without events were censored at their last evaluation date.

Cumulative incidence of second relapse (CIR), treatment-related mortality (TRM), and chronic GVHD (cGVHD) were calculated considering a subsequent relapse (with TRM as a competing event), death in the absence of second relapse (with second relapse and second malignant neoplasm as competing events), and date of diagnosis of cGVHD as events, respectively.

Chronic GVHD-free and leukemia-free survival (GLFS) was calculated considering the diagnosis of cGVHD (any grade) as an additional event to those applied for the analysis of LFS (39).

Follow-up time was defined as time from transplantation to death or last contact.

The Kaplan–Meier and log-rank tests were applied for the generation and comparison of survival curves. Cumulative incidence curves were generated and compared according to the method of Fine and Grey. The Cox proportional hazard regression model with estimate of hazard ratios for any individual risk factor was applied for multivariate analysis. The R software platform was used for statistical analyses.

Data collection was completed on 6 April 2022.

## Results

### Characteristics of patients and donors

Between January 2015 and March 2022, 76 patients included in the “SEHOP/PETHEMA 2015” registry received an SCT as consolidation therapy in CR2. A total of 25 patients underwent haploidentical donor transplantation and the remaining 51 were transplanted from an HLA-matched donor.

One patient with a late isolated extramedullary relapse (*KMT2A*-rearranged) was misclassified as HR relapse, achieved MRD-negative CR2 after reinduction, and underwent SCT from a matched unrelated donor. Six additional patients included in this study (five with late isolated or combined BM relapse of BCP ALL and MRD good response after reinduction and one with early isolated extramedullary relapse without an available matched donor) underwent SCT despite not fulfilling the criteria specified in the guidelines to receive a transplantation (Supplementary Table S4).

One patient directly underwent haploidentical donor SCT after reinduction in CR2 (MRD-negative) because of severe toxicity during reinduction and another underwent SCT from an unrelated donor after two (instead of three) consolidation blocks.

One patient with an early isolated BM relapse of BCP ALL was misclassified as SR relapse, failed to respond to SR reinduction, and achieved CR2 after salvage third-line therapy before SCT. Twelve additional patients who failed to respond to reinduction or had persistent MRD positivity during consolidation received off-guideline individualized “rescue” therapy and were included in this study after achieving CR2 before SCT (Supplementary Table S5).

One patient with HR relapse was included in the NCT02393859 study and received one cycle of blinatumomab as third consolidation before SCT.

Table 2 presents the clinical characteristics of patients and donors. Mean age at transplantation was 9 years (range: 1–19). The patients were equally distributed across the haploidentical and the HLA-matched donor groups according to the following variables: age, leukemia immunophenotype, time to relapse, site of relapse, risk group allocation at relapse, and MRD before transplantation. In total, 66 patients (86.8%) had BCP and 10 (13.2%) had T-cell immunophenotye ALL; 15 (19.8%) had very early, 35 (46%) early and 26 (34.2%) had late relapses; 47 (61.8%) had isolated BM, 21 (27.6%) combined BM, and 8 (10.5%) had isolated extramedullary relapse; 41 (53.9%) and 35 patients (46.1%) were allocated to the HR and SR groups at relapse, respectively; MRD before transplantation was ≥0.01% in 9 patients (12.2%) and <0.01% in 65 patients (87.8), and for 2 patients, data were not available data.

**Table 2:** Characteristics of patients and donor-recipient HLA matching.

	Haploidentical donor		HLA-matched donor		*p*
	*n* = 25	%	*n* = 51	%	
**Age at SCT (years)** ^a^
≥Mean	12	48	27	52.9	.6855
<Mean	13	52	24	47.1	
**Immunophenotype**
B-cell precursor	23	92	43	84.3	.3517
T	2	8	8	15.7	
**Time to relapse** ^b^
Very early	5	20	10	19.6	1
Early	12	48	23	45.1	
Late	8	32	18	35.3	
**Site of relapse**
Isolated BM	18	72	29	56.9	.3323
Combined BM	6	24	15	29.4	
Isolated EM	1	4	7	13.7	
**Risk-group at relapse**
High	16	64	25	49	.2183
Standard	9	36	26	51	
**MRD before SCT** ^c,d^
≥0.01%	2	8.3	7	14	.4851
<0.01%	22	91.7	43	86	
No data	1		1		
**HLA matching (matched alleles)** ^d^
9/10	na	na	8	16	na
10/10	na	na	36^e^	72	na
Umbilical cord blood (5–6/6)	na	na	6	12	na
No data	na	na	1		na

BM, bone marrow; EM extramedullary; HLA, human leukocyte antigen; MRD, minimal residual disease; na, not applicable; SCT, allogeneic stem cell transplantation;.

^a^ Mean age 9 years (range: 1 to 19).

^b^ Very early relapse: <6 months after the end of first-line treatment and <18 months after primary diagnosis. Early relapse: <6 months after the end of first-line treatment but ≥18 months after primary diagnosis. Late relapse: ≥6 months after the end of first-line treatment.

^c^ Five patients had an MRD ≥0.1% (four in the haploidentical group and one in the HLA-compatible donor group).

^d^ Patients without available data were excluded from the analysis of the corresponding variable.

^e^ Sixteen related and 20 unrelated donors.

All except one patient younger than 2 years (high risk) were classified as intermediate risk according to the validated pediatric disease risk index (40).

In the HLA-matched donor group, 16 patients (32%) were transplanted from a matched sibling donor, 28 (56%) from an unrelated donor, and 6 (12%) from an umbilical cord blood unit.

If we focus on BCP ALL, after excluding 10 cases with T-cell immunophenotype, patients remained equally distributed according to age, time to relapse, site of relapse, risk-group allocation at relapse, and MRD before transplantation (Supplementary Table S6).

### Transplantation procedure

The combination of thiotepa, busulfan, and fludarabine was the most frequently used conditioning regimen, particularly in the group of haploidentical donor transplantation (Table 3). Most patients in the haploidentical donor group (88%) received chemotherapy-based conditioning, and the proportion of patients who received a total body irradiation (TBI)-based conditioning regimen was significantly higher in the HLA-matched donor group (50% vs. 12%; *p* = .0013). More patients in the haploidentical donor group received peripheral blood (80% vs. 48%), although this was the most prevalent stem cell source in both cohorts (Table 3).

**Table 3:** Transplantation characteristics.

	Haploidentical donor		HLA-matched donor		*P*
	*n* = 25	%	*n* = 51	%	
**Conditioning** ^a^
TT + Bu + Flu	15	60	16	32	**.** **0203**
Bu + Cy ± TT	0	0	5	10	0.1017
TBI + VP16	1	4	8	16	0.1317
TBI + Cy	0	0	8	16	**.** **0343**
TLI + TT + Flu + L-PAM	6	24	2	4	**.** **0082**
TBI + TT + Cy	0	0	5	10	.1017
Other^b^	3	12	6	12	1
No data	0		1		
**TBI-based conditioning** ^a^
Yes	3	12	25	50	**.** **0013**
No	22	88	25	50	
No data	0		1		
**Stem cell source^a^**
Peripheral blood	20	80	24	48	**.** **0177**
Bone marrow	5	20	20	40	
Umbilical cord blood	0		6	12	
No data	0		1		
***Ex vivo* graft manipulation^a,c^**
No manipulation	10	41.7	47	92.2	**<** **.** **001**
αβ T-cell and CD19 + depletion	8	33.3	0	0	**<** **.** **001**
CD45RA + depletion	5	20.8	4	7.8	.1063
CD3 + and CD19 + depletion	1	4.2	0	0	.1422
No data	1		0		
**CD34 + cell dose infused^a,d^**
≥mean	12	50	23	50	1
<mean	12	50	23	50	
≥2 × 10^6^ CD34+/kg	23	95.8	40 (35^e^)	86.9 (87.5^e^)	0.24 (0.26)
≥5 × 10^6^ CD34+/kg	15	62.5	27 (24^e^)	58.7 (60^e^)	0.75 (0.84)
No data	1		5		
**GVHD prophylaxis^a^**
Cyclosporine + methotrexate	0	0	27	54	**<** **.** **001**
Cyclosporine	8	33.3	6	12	**.** **0283**
Cyclosporine + MF	5	20.8	1	2	**.** **0055**
Tacrolimus + methotrexate	0	0	5	10	.1086
Tacrolimus + MF	4	16.7	0	0	**.** **003**
Tacrolimus	1	4.2	3	6	.7441
MF	2	8.3	2	4	.4403
Methotrexate	0	0	1	2	.4855
Cyclosporine + Prednisolone	0	0	1	2	.4855
None^f^	4	16.7	4	8	.261
No data	1		1		
**Serotherapy^a^**
Yes	4	16.7	32	62.7	**<** **.** **001**
No	20	83.3	19	37.3	
No data	1		0		

Bu, busulphan; Cy, cyclophosphamide; Flu, fludarabine; GVHD, graft vs. host disease; L-PAM, melphalan; na, not applicable; MF, mycophenolate mofetil; TBI, total body irradiation; TLI, total lymphoid irradiation; irradiation; TT, Thiotepa; VP16, etoposide.

^a^ Patients without any records or data were excluded from the analysis of the corresponding variable.

^b^ Other conditioning regimens: Bu + TT; Bu + Flu; Cy + VP16; TBI + Flu ± Cy or TT; TBI + Cy + VP16; TBI + TT.

^c^ Patients without graft manipulation (T-cell-replete grafts) in the haploidentical donor group received post-transplant cyclophosphamide.

^d^ Mean CD34+ cell dose infused was 5.3 × 10^6^/kg (range 0.35–14.6).

^e^ Excluding 6 patients who received umbilical cord blood transplantation (*n* = 44).

^f^ One and three patients in the haploidentical donor group received no pharmacologic GVHD prophylaxis after αβ T-cell and CD45RA+ depletion, respectively; 4 in the HLA compatible donor group received no pharmacologic GVHD prophylaxis after CD45RA+ graft depletion.

Ten out of 25 patients in the haploidentical donor group received post-transplant cyclophosphamide after T-cell replete haploidentical graft infusions, 14 patients received *ex vivo* T-cell-depleted allografts (8 αβ T-cell, 5 CD45RA+, and 1 CD3+ depletion), and data were not available for one patient.

Half of the patients in both groups received allografts with a CD34+ cell dose above a mean dose of 5.3 × 10^6^/kg (range 0.35–14.6).

Four patients in each group received no pharmacological GVHD prophylaxis after T-cell depleted SCT, and cyclosporine alone or in combination with methotrexate was the most prevalent approach for the remaining patients. Serotherapy was more frequently applied in the HLA-matched donor group (Table 3).

The distribution of BCP ALL patients according to all these transplantation variables is shown in Supplementary Table S7.

### Early complications

Graft failure occurred in four (16%) and two patients (4%) in the haploidentical and the HLA-matched donor groups, respectively; this difference was not statistically significant.

We could not find a correlation between CD34 cell doses and the incidence of primary or secondary graft failure(*p* = 0.642) ; mean CD34+ cell dose of patients with and without graft failure was 5,85 and 5,2, respectively (table S8)” instead of “We could not find a correlation between CD34 cell doses and the incidence of primary or secondary graft failure; mean CD34+ cell dose of patients with and without graft failure was 5,85 and 5,2, respectively (*p* = 0.642) (table S8).

Similarly, there were no differences in the incidence of grade 1 and grade ≥2 acute GVHD (aGVHD), grade ≥3 infectious complications, any grade SOS, and other CTCAE grade ≥3 early complications (Table 4). Supplementary Table S9 includes information about early complications in BCP ALL patients.

**Table 4:** Early complications after stem cell transplantation and chronic GVHD.

	Haploidentical donor		HLA-matched donor		*p*
	*n* = 25	%	*n* = 51	%	
Graft failure^a^
Yes	4	16	2	4	.071
No	21	84	48	96	
No data	0		1		
aGVHD
Grade 1	3	12	3	5.9	.6566
Grade ≥2	9	36	19	37.2	
No	13	52	29	56.9	
Grade ≥3 infections
Yes	15	60	26	51	.4586
No	10	40	25	49	
SOS (any grade)
Yes	2	8	10	19.6	.1923
No	23	92	41	80.4	
Other CTCAE grade ≥3^a^
Yes	11	45.8	20	41.7	.7364
No	13	54.2	28	58.3	
No data	1		3		
cGVHD^a,b^
Yes	1	4.8	7	17.1	.1711
No	20	95.2	34	82.9	
No data	1		1		

aGVHD, acute graft vs. host disease; cGVHD, chronic graft vs. host disease; CTCAE, Common Terminology Criteria for Adverse Events version 3.0; SOS, sinusoidal obstructive syndrome (any grade).

^a^ Patients without any records or data were excluded from the analysis of the corresponding variable.

^b^ Proportions of patients with any-grade cGVHD among those surviving >100 days after transplantation (22 in the haploidentical and 42 in the HLA-matched donor groups).

Nineteen patients died of treatment-related complications, six (24%) in the haploidentical group and 13 (25.5%) in the HLA-matched donor group. Eight of these deaths in remission occurred in patients diagnosed with grade 3/4 aGVHD, severe infection was reported as the cause of death in five patients (three invasive fungal and two cytomegalovirus and/or other viral infections), severe SOS was present in five (all in the HLA-matched donor group), and one patient died as a consequence of post-transplant lymphoproliferative disease. Of note, two of these treatment-related deaths occurred in children undergoing STC (one matched unrelated and one haploidentical donor) without a scheduled indication (late isolated or combined bone marrow relapse with MRD good response after induction) (Supplementary Table S4).

Six out of 16 patients transplanted from an HLA-matched related donor survived in CR2 after a median follow-up of 64 months (range 16–79 months), seven had a subsequent relapse, and three died in remission.

### Analysis of prognostic factors and outcome

Five (20%) and nine (17.6%) patients relapsed and died after haploidentical and HLA-matched donor SCTs, respectively.

**Figure 1:**
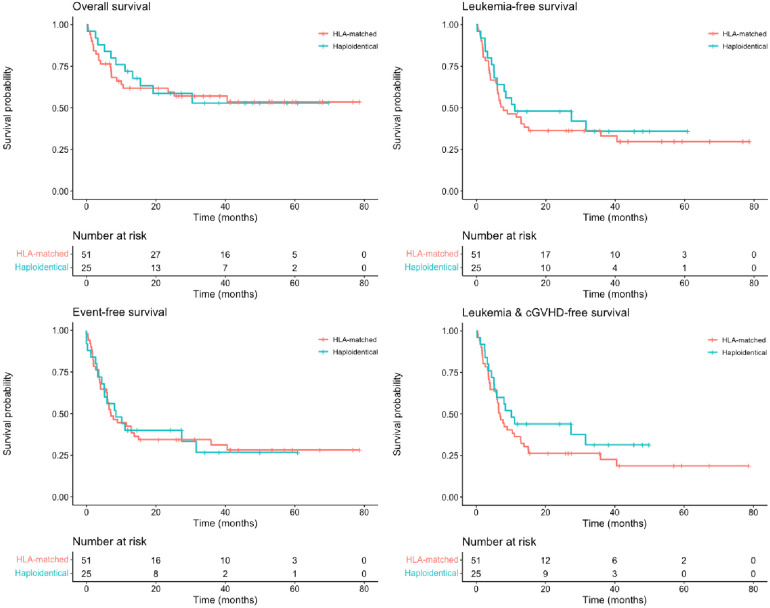
Two-year overall survival (58.7% vs. 59.5%; *p* = .8), leukemia-free survival (48% vs. 36.4%; *p* = .5), event-free survival (40% vs. 34.4%; *p* = 1), and chronic graft vs. host disease–free and leukemia-free survival (44% vs. 26.3%; *p* = .3) among patients undergoing transplantation from haploidentical (blue lines) and HLA-matched (red lines) donors.

**Figure 2:**
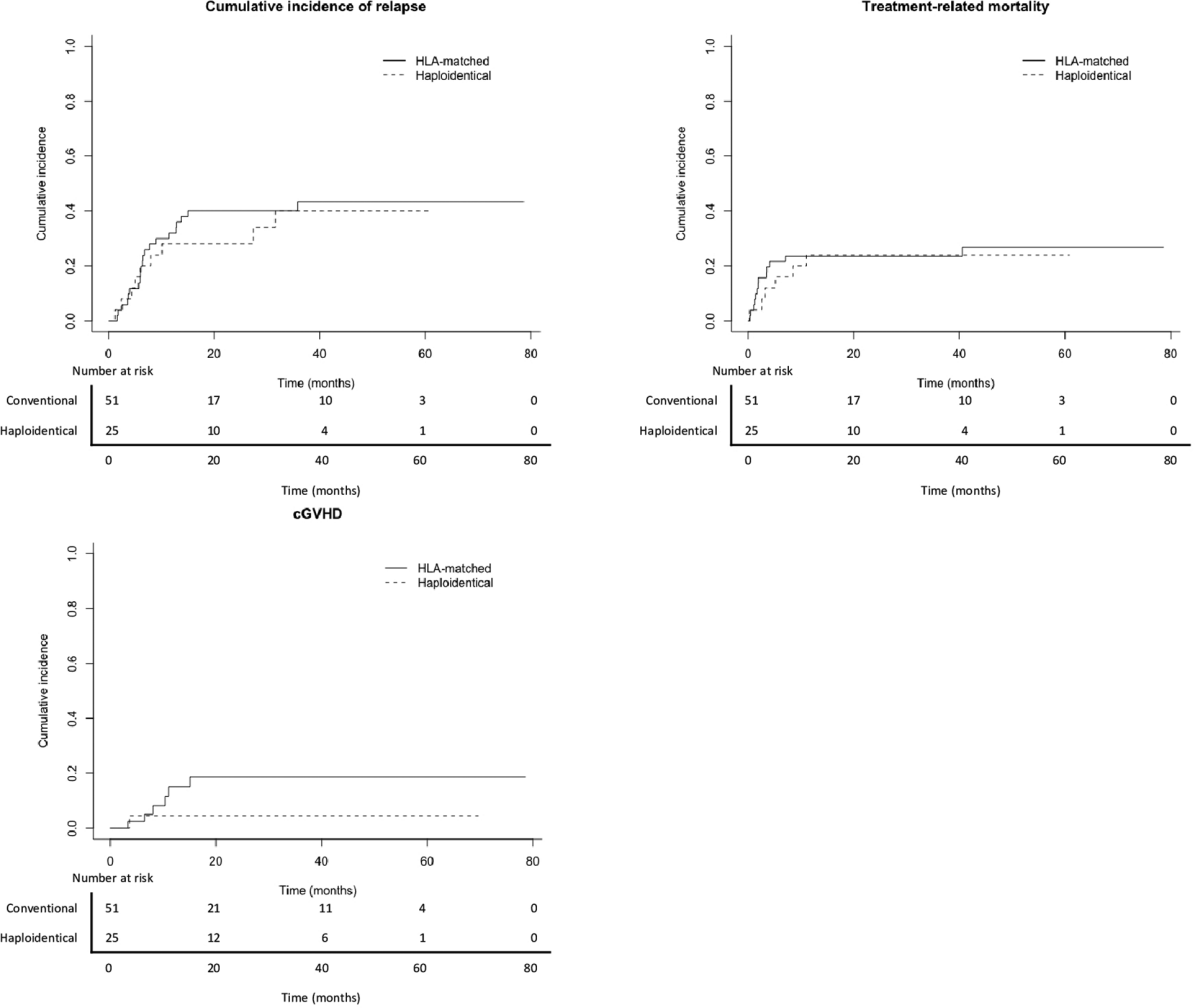
Two-year cumulative incidence of second relapse (28% vs. 40.9%; *p* = .69), treatment-related mortality (24% vs. 23.6%; *p* = .83), and chronic graft vs. host disease (4.5% vs. 18.7%; *p* = .2) among patients undergoing transplantation from haploidentical (dashed lines) and HLA-matched donors (solid lines).

After a median follow-up of 22 months (0–78.7 months), 21 (0–71) in the haploidentical, and 24 (0–80) in the HLA-matched donor groups, we found no significant differences in the estimated rate of 2-year OS (58.7% vs. 59.5%; *p* = .8), LFS (48% vs. 36.4%; *p* = .5), and EFS (40% vs. 34.4%; *p* = 1) among patients undergoing transplantation from haploidentical or HLA-matched donors (Table 5 and Figure 1). Moreover, there were no significant differences in the 2-year CIR (28% vs. 40.9%; *p* = .69) and TRM (24% vs. 23.6%; *p* = .83) (Table 5 and Figure 2).

**Table 5:** Overall results: probability (%) and 95% CI.

	Haploidentical donor	HLA-matched donor	*p*
OS	58.7% (41.9–82.2)	59.5% (47.2–75)	.8
LFS	48% (31.9–72.2)	36.4% (25.2–52.5)	.5
EFS	40% (24.7–64.6)	34.4% (23.4–50.4)	1
CIR	28% (12.1–46.5)	40.9% (26.3–53.4)	.69
TRM	24% (9.4–42.2)	23.6% (13–36)	.83
Cumulative incidence of cGVHD	4.5% (0.2–19.4)	18.7% (7.3–34.3)	.2
GLFS	44% (28.3–68.5)	26.3% (16.5–41.9)	.3

cGVHD, chronic graft vs. host disease; GLFS, cGVHD-free and leukemia-free survival; CIR, cumulative incidence of relapse; EFS, event-free survival; LFS, leukemia-free survival; OS, overall survival; TRM, treatment-related mortality.

Among 64 patients who survived >100 days after SCT, 1 patient (4.8%) in the haploidentical and 7 (17.1%) in the HLA-matched donor cohorts were diagnosed with mild or moderate chronic GVHD (cGVHD), respectively (Table 4).

There was no difference in the 2-year cumulative incidence of cGVHD (4.5% vs. 18.7%; *p* = .2) and GLFS (44% vs. 26.3%; *p* = .3) between the haploidentical and the HLA-matched donor groups (Table 5 and Figures 1, 2).

We analyzed the impact of the following factors on LFS: leukemia immunophenotype (BCP vs. T-cell immunophenotype), risk-group allocation at relapse (SR vs. HR), MRD before SCT (<0.01% vs. ≥0.01%), conditioning regimen (TBI-based vs. chemotherapy-based), CD34+ cell dose (above/below the mean), grade 1 and 2 aGVHD, and any-grade cGVHD.

An MRD < 0.01% before SCT (37.1% vs. 0%; *p* = .02) and the occurrence of any-grade cGVHD (85.7% vs. 35.3%; *p* = .01) were associated with a significantly higher LFS (Table 6).

**Table 6:** Factors influencing leukemia-free survival: univariate analysis.

	N. of patients	Events	Probability (%)	95% CI	*P*-value
Immunophenotype
B-cell precursor	66	41	43.1	32.6–57.1	.2
T	10	8	20	5.79–69.1	
Risk group at relapse
Standard risk	35	19	50.5	36.2–70.4	.08
High risk	41	30	31.4	19.9–49.5	
MRD before SCT
<0.01%	65	38	37.1	26.1–52.8	**.** **02**
≥0.01%	9	9	0		
No data	2				
Conditioning regimen
TBI-based	28	16	48.6	33–71.6	.2
Chemotherapy-based	47	32	35.8	24.3–52.7	
No data	1				
CD34+ cell dose infused
<mean	35	22	48.4	34.3–68.2	.8
≥mean	35	22	35.8	22.8–56.2	
No data	6				
Grade 1/2 aGVHD
Yes	15	41	49.9	29.2–85.2	.4
No	61	8	37.7	27.3–52.1	
Any-grade cGVHD
Yes	7	1	85.7	63.3–100	**.** **01**
No	69	48	35.3	25.5–48.8	

aGVHD, acute graft vs. host disease; cGVHD, chronic graft vs. host disease; MRD, minimal residual disease; N., numbers; SCT, stem cell transplantation; TBI, total body irradiation.

In multivariate analysis, the occurrence of cGVHD (HR = 0.09; *p* = .02) had a significant protective impact, while an MRD ≥ 0.01% (HR = 2.59; *p* = .01) had an unfavorable impact (Table 7).

**Table 7:** Factors influencing leukemia-free survival: multivariate analysis.

	Hazard ratio (95% CI)	*p*-value
MRD before SCT ≥ 0.01%	2.59 (1.23–5.46)	.0121
Any grade cGVHD	0.09 (0.01–0.7)	.0210

cGVHD, chronic graft vs. host disease; MRD, minimal residual disease; SCT, stem cell transplantation; TBI, total body irradiation.

After excluding patients with T-cell immunophenotype, only MRD ≥ 0.01% before SCT remained significant. A separate analysis of prognostic factors and outcome of patients with BCP ALL is provided as Supplementary material (Supplementary Tables S10–S12 and Figures S1, S2).

## Discussion

The “SEHOP/PETHEMA 2015” guidelines and registry were developed by the Leukemia Working Group of the SEHOP in order to provide a common approach for the diagnosis and treatment of pediatric patients with first relapse of ALL in Spain. A prospective data registry was generated, which allowed us to analyze treatment results. In this study, we focused on the analysis and comparison of treatment outcomes of 76 children undergoing SCT in CR2 from haploidentical vs. HLA-matched donors. We found no significant differences in the estimate of OS, LFS, EFS, CIR, and TRM between both groups.

Noteworthily, the distribution of patients was well balanced according to defined prognostic factors at relapse and MRD before transplantation. Leukemia immunophenotype, time to relapse, and site of relapse are well-recognized prognostic factors after first relapse of ALL in children and, according to these factors, patients are often stratified as SR- or HR relapse (29, 41–45). The distribution of patients according to the site of relapse is comparable to that presented in other series. On the other hand, we had higher (46%) and lower (34.2%) proportions of early and late relapses, respectively (2, 3, 46, 47), and the proportion of patients with T-cell immunophenotype (13.2%) was relatively low (1, 13, 27, 30–32, 46, 47). As previously described, we found no significant impact of leukemia immunophenotype and risk group allocation at relapse in LFS after SCT (13, 30).

All patients in our study were in CR2 at the time of SCT and, accordingly, these prognostic factors, which are determinant at diagnosis of relapse, seem to lose their impact on final outcome, provided that patients achieve a new remission and undergo SCT as consolidation treatment (3). This is supported by previous studies reporting substantial differences in the final outcomes of children who do and do not undergo SCT after HR first relapse of ALL (30). With a few exceptions (46), MRD before SCT has been related to final outcomes in children with ALL (2, 3, 13, 30, 47, 48). Some studies define as positive an MRD ≥ 10^−3^ before SCT when assessed by using flow cytometry or by real-time quantitative polymerase chain reaction for clonal gene rearrangements (30, 46–48). Given that only 5 out of 74 patients with available data in our series had MRD ≥ 10^−3^, we applied an inferior threshold of >10^−4^ and, according to this level, the proportions of patients with positive MRD were not different in the haploidentical and the HLA-matched donor groups. In our study, an MRD ≥ 0.01% before SCT had a negative impact in LFS both in the univariate and in the multivariate analyses, which was in accordance with the results presented by the ALL-REZ BFM Study Group (3).

The conditioning regimen has a significant impact on treatment outcome after SCT in children with ALL. Although excellent outcomes were reported from patients with ALL undergoing transplantation from an HLA-matched donor after conditioning with TBI and etoposide (1, 31, 32), the cumulative incidence of long-term serious adverse events, including second malignant neoplasms, was more frequently associated with the use of TBI, particularly in younger patients (49, 50). However, TBI-based conditioning regimen before SCT has been clearly associated with a lower CIR and higher LFS (13, 51). An international, randomized, phase III study in children with ALL undergoing transplantation from HLA-matched donors (ALL SCTped 2012 FORUM Study) recently demonstrated a high superiority of TBI plus etoposide as a conditioning regimen when compared with other chemoconditioning (non-TBI) regimens in terms of OS (primary objective) and EFS. In fact, in this study, randomization was terminated early after an interim analysis from an independent Data Monitoring Committee demonstrating a lower risk of relapse and TRM with the TBI arm (46). Similarly, a TBI-containing regimen had a positive impact on LFS in children undergoing haploidentical donor SCT after αβ T-cell and B-cell depletion (13). In our study, different conditioning regimens were recorded, and the combination of thiotepa, busulfan, and fludarabine was the most prevalent modality. This regimen is frequently reported as preparative conditioning treatment before haploidentical donor SCT (8, 16, 21, 25). In total, 47 out of 75 patients (62.7%) with available data in our study were conditioned without TBI, and the proportion of patients conditioned with non-TBI regimens was higher in the haploidentical donor group.

Although patients conditioned with TBI had a higher rate of LFS (48.6% vs. 35.8%), this difference was not statistically significant (*p* = .2; Table 6 and Supplementary Figure S3).

As expected, more patients in the haploidentical donor group received peripheral blood, although this was the most prevalent stem cell source in both cohorts, which probably reflected donor choice (Table 3).

Graft rejection remains one of the major challenges for success after haploidentical donor SCT, and stem cell content within the graft influences the risk of graft failure. In total, the mean CD34+ cell dose in our study was lower than that presented in other series (8, 10, 12–14, 17, 21, 22, 24). This might explain a higher rate of graft failure, particularly in the haploidentical donor group. However, the proportions of patients who received CD34+ cell doses above or below the mean value were equally distributed between both groups, and more patients in the haploidentical donor group experienced graft failure (16% vs. 4%), although this difference was not significant (Table 4).

Similarly, there was no difference in the proportions of patients experiencing aGVHD between both groups, which were similar to that commonly reported after haploidentical and HLA-matched donor SCTs (1, 8–14, 16–18, 21, 22, 24–27, 31, 32, 46). The use of anti-T-lymphocyte globulin (serotherapy) is often scheduled during conditioning in order to prevent graft rejection and GVHD (1, 2, 10, 13, 14, 21, 22, 26, 52). In our study, more patients in the HLA-matched than in the haploidentical donor group received serotherapy (62.7% vs. 16.7%; *p* < .001), which reflected a relatively high proportion of unrelated donors in the HLA-matched donor group (56%) and a significant proportion of patients in the haploidentical donor group who received post-transplant cyclophosphamide (41.7%). Moreover, all our patients were diagnosed with ALL relapse and underwent transplantation after January 2015 and, consequently, modern *ex vivo* graft manipulation strategies such as αβ T-cell/CD19+ and CD45RA+ depletion were the most frequently applied (16). The use of these novel and more sophisticated methods of T-cell depletion probably prevented the administration of serotherapy in many patients within the haploidentical donor group. Even when more patients in the conventional donor group received serotherapy, this did not translate into significant lower rates of graft failure and aGVHD. In contrast to previous reports, we did not find an association between the occurrence of grade 1/2 aGVHD and LFS (30, 47, 48).

The 2-year cumulative incidence of cGVHD was 18.7% in the HLA-matched donor group and 4.5% in the haploidentical donor cohort. This result is in contrast to that of previous studies reporting a cumulative incidence of cGVHD higher than 25% in children undergoing haploidentical donor SCT but is in line with the results of other studies after *ex vivo* T-cell depletion (Supplementary Table S13) (2, 10, 13, 16, 17, 20, 26). As a consequence of a lower cumulative incidence of cGVHD, we found a better GLFS rate in the haploidentical group than in the HLA-matched donor group (44% vs. 26.3%), although this difference was not significant (*p* = .3; Figure 1).

Chronic GVHD is consistently associated with a lower risk of relapse after both conventional and haploidentical donor transplantations (8, 11, 16, 21, 23, 53). In this study, the occurrence of any-grade cGVHD had a remarkable positive impact on LFS.

The incidence of severe infections, SOS (any grade), and other grade ≥3 adverse events was not different between both SCT cohorts. As expected, the common causes of nonrelapse mortality were related to aGVHD, infection, and toxicity (SOS) (45, 46). The estimation of 2-year TRM in our series was 24% and 23.6% in the haploidentical and the HLA-matched donor groups, respectively. This difference was not statistically significant, and they were higher than those reported in most recent studies, which might be explained in part by the fact that all our patients underwent transplantation in CR2. Consequently, they were heavily pretreated, while other series of SCT included patients with different hematological malignancies and many of them were transplanted as part of first-line consolidation treatment (Supplementary Table S13) (1, 2, 13, 27, 46, 47, 51). However, our TRM results were worse than those reported in two recent studies from patients with HR relapse undergoing SCT in CR2 (30, 46). The FORUM study had some clear advantages: it was run as a prospective well-controlled trial with stringent criteria for donor selection, stem cell source, and other aspects related to transplantation procedures such as MRD monitoring, GVHD prophylaxis, and conditioning regimen (46). We did not analyze the so-called center effect, which has been shown to be the determinant in treatment outcomes after alternate donor SCT (24). In order to reduce TRM, we will encourage the participation of Spanish transplantation centers within the FORUM network activities and the establishment and application of nationwide harmonized transplantation operating procedures with standardized supportive care protocols (32).

Subsequent relapse represents a common cause of treatment failure in pediatric patients undergoing SCT for ALL (27, 32, 46, 47). Our CIR rates (28% and 40.9% in the haploidentical and HLA-matched donor groups, respectively) were higher than those previously reported after SCT for ALL in children, which translated into lower OS, EFS, and LFS rates. As previously mentioned, it should be considered that, in contrast to our series, these studies included many patients undergoing SCT in first remission, and, in most of them, remission status influenced the final outcome (1, 2, 13, 26, 27, 31, 32). However, if we focus on specific data from children with ALL undergoing SCT in CR2, we can see that our estimated CIR is in line with that from previous studies, particularly after a non-TBI conditioning regimen (Supplementary Table S13) (24, 26, 27, 30, 46, 51). In order to prevent relapse after SCT, we will need to reduce the tumor burden before SCT through the application of modern leukemia treatment approaches, including specific monoclonal antibodies and chimeric antigen receptor T-cell therapy, improve the efficacy of the conditioning regimens, and explore the role of post-transplant interventions such as early withdrawal of immunosuppression and adoptive immunotherapy strategies (3, 16, 19, 24, 31, 48, 54). In this context, given the donor availability and proximity, haploidentical SCT offers an ideal platform for the design of such trials (4, 14, 20, 24). Four patients in each cohort received no GVHD pharmacological prophylaxis after *ex vivo* T-cell depletion. In this scenario, the administration of certain immunotherapy approaches such as bispecific monoclonal antibodies after transplant emerges as an attractive option, able to take advantage of the donor alloreactive immune system, instead of leaning on an often-exhausted host T-cell compartment before transplantation (22).

Given the retrospective design of this multicenter study, the low number of patients, the lack of data regarding variables such as leukemia cytogenetic risk profile, donor age and gender, donor–patient relationship, and donor/receptor KIR mismatch, and given the heterogeneity of the two cohorts of patients included in terms of conditioning regimens, stem cell source, GVHD pharmacological prophylaxis, and the application of serotherapy, we did not intend to study the impact of such variables on outcomes, nor the impact of stem cell source on the incidence of acute and cGVHD, and we did not analyze other specific transplant-related outcome parameters such as engraftment kinetics and immune reconstitution.

With regard to sample size, we estimated that in order to demonstrate a 20% improvement in LFS and GLFS after a median follow-up of 5 years at a power of 80% with a *p*-value of .05, based on our baseline event rate and assuming a 0 censoring rate and a distribution of 33% vs. 67% in both groups, we would need 352 and 235 patients recruited in the haploidentical donor group and 716 and 478 in the HLA-compatible donor group, respectively (1,068 and 713 events in total).

The proportion of patients conditioned with TBI was lower in the haploidentical donor group (12% vs. 50%; *p* = .0013, Table 3); moreover, although nonsignificant, the proportion of patients stratified as HR at relapse was higher in the haploidentical donor group (64% vs. 49%; *p* = .2183; Table 2). This unequal distribution limited the ability of the study to identify superior outcomes with haploidentical vs. HLA-matched donor SCT.

Another important limitation is determined by the short median follow-up period. Moreover, we acknowledge that seven patients (two in the haploidentical and five in the HLA-matched donor cohort) underwent transplantation without fulfilling specified criteria, and 13 patients (six in the haploidentical and seven in the HLA-matched donor groups) received additional therapy to achieve MRD-negative CR2 before SCT, and this may have influenced the final outcome.

However, all patients included in this study were prospectively recorded in a nation-wide (SEHOP-PETHEMA 2015) registry for children with first relapse of ALL and underwent SCT in CR2. Our results may serve as a historical control for future studies exploring alternate approaches for children after first relapse of ALL.

In summary, we found no difference in treatment outcomes among pediatric patients with ALL in CR2 undergoing SCT from haploidentical and HLA-matched donors. These results support the role of haploidentical donors as an alternative to HLA-compatible donors in this population.

## Data Availability

The raw data supporting the conclusions of this article will be made available from the corresponding author upon reasonable request.

## Glossary

aGVHD, acute graft vs. host disease; ALL, acute lymphoblastic leukemia; ALL-REZ BFM 2002, Acute Lymphoblastic Leukemia Relapse Berlin-Frankfurt-Münster 2002; BCP, B-cell precursor; BFM, Berlin–Frankfurt–Muenster; BM, bone marrow; cGVHD, chronic graft vs. host disease; CIR, cumulative incidence of second relapse; CR2, second complete remission; CTCAE, Common Terminology Criteria for Adverse Events; EFS, event-free survival; GLFS, chronic graft vs. host disease–free and leukemia-free survival; GVHD, graft vs. host disease; HLA, human leukocyte antigen; HR, high risk; LFS, leukemia-free survival; MRD, minimal residual disease; NK, natural killer; OS, overall survival; SCT, Allogeneic stem cell transplantation; SEHOP, Spanish Society of Pediatric Hematology and Oncology; SOS, sinusoidal obstructive syndrome; SR, standard risk; TBI, total body irradiation; TRM, treatment-related mortality.
