# Supplementary material for: Haploidentical vs. HLA-matched donor hematopoietic stem-cell transplantation for pediatric patients with acute lymphoblastic leukemia in second remission: A collaborative retrospective study of the Spanish Group for Bone Marrow Transplantation in Children (GETMON/GETH) and the Spanish Childhood Relapsed ALL Board (ReALLNet)
Source: Front Pediatr. 2023 Mar 20;11:1140637. doi: 10.3389/fped.2023.1140637 (PMC10067875; doi:10.3389/fped.2023.1140637)
Supplement: Supplementary file 1 [file Datasheet1.docx]

Supplementary Material

**Haploidentical *versus* HLA-matched donor hematopoietic stem-cell transplantation for pediatric patients with acute lymphoblastic leukemia in second remission: A collaborative retrospective study of the Spanish Group for Bone Marrow Transplantation in Children (GETMON/GETH) and the Spanish Childhood Relapsed ALL Board (ReALLNet)**

Celia Moreno ^1^, Eduardo Ramos-Elbal ^1^, Pablo Velasco ^2^, Yurena Aguilar ^3^, Berta González ^4^, Carolina Fuentes ^5^, Águeda Molinos ^6^, Pilar Guerra-García ^4,^ ^7^, Pilar Palomo ^8^, Jaime Verdu ^9^, Rosa María Adán ^10^, José Manuel Vagace ^11^, Mónica López Duarte ^12^, Alexandra Regueiro ^13^, María Tasso ^14^, José Luis Dapena ^15, 16^, José Antonio Salinas ^17^, Samuel Navarro ^17^, Francisco Bautista ^18^, Álvaro Lassaletta ^19^, Francisco Lendínez ^20^, Susana Rives ^15, 16^, Antonia Pascual ^21^, Antonia Rodríguez ^22^, José María Pérez-Hurtado ^6^, José María Fernández ^5^, Antonio Pérez-Martínez ^4^, Marta González-Vicent ^19^, Cristina Díaz de Heredia ^2^, José Luis Fuster* ^1, 23^

*** Correspondence:** José Luis Fuster, Hospital Clínico Universitario Virgen de la Arrixaca, Ctra. Madrid-Cartagena s/n, 30120, El Palmar, Murcia (Spain). Phone: (34) 968369298. Email: [josel.fuster@carm.es](mailto:josel.fuster@carm.es). ORCID: 000-0002-4881-9440

TABLE OF CONTENTS Page

Table S1 ……………………………………………………………………..…… 2

Table S2 …………………………………………………………………..……… 2

Table S3 ………………………………………………………………………..… 3

Table S4 ………………………………………………………………………….. 4

Table S5 ………………………………………………………………………..… 5

Table S6 ………………………………………………………………………..… 6

Table S7 ………………………………………………………………………..… 7

Table S8 and S9 ……………………………………………………………..…… 8

Table S10, S11 and S12 ………………………………………………………..… 9

Table S13 ………………………………………………………………………... 10

Figure S1 …………..…………………………………………………………...... 12

Figure S2 ……………………………………………………………………….... 13

Figure S3 ………………………………………………………………………… 14

REFERENCES ………………………………………………………………….. 15

**Table S1. Definition of relapse**

|  | **Bone marrow ^1^** | **M1**  **(< 5% blasts)** | **M2**  **(≥ 5% and < 25% blasts)** | **M3**  **(≥ 25% blasts)** |
| --- | --- | --- | --- | --- |
| **Extramedullary disease ^2^** | **No** | No relapse | Repeat bone marrow evaluation | Isolated bone marrow relapse |
|  | **Yes** | Isolated extramedullary relapse | Combined (bone marrow and extramedullary) relapse | |

^1^ The percentage of leukemic blasts was established by conventional cytology.

^2^ Central nervous system relapse is defined as the identification of leukemic lymphoblasts in the cerebrospinal fluid with > 5 nucleated cells/µl or the presence or tumor lesions or evidence of meningeal infiltration by imaging or biopsy; testicular relapse is defined as a confirmatory testicular biopsy in patients with painless testicular enlargement; other extramedullary sites of relapse are diagnosed by radiological measures with confirmation by biopsy.

**Table S2. Treatment protocol before stem cell transplantation**

| **High risk relapse** |  |  |
| --- | --- | --- |
| **Induction “HIA” (R3)** |  |  |
| Dexamethasone | 10 mg/m^2^/ 12 h. | Days 1 to 5 of week 1 and week 3 |
| Vincristine | 1.5 mg/m^2^ | Days 3 of weeks 1, 2 ,3 and 4 |
| Mitoxantrone | 10 mg/m^2^ | Days 1 and 2 of week 1 |
| PEG-asparaginase | 1,000 U/m^2^ | Day 3 of week 1 and 3 |
| TIT ^1^ |  | Day 1 of weeks 1 and 2 |
| **Consolidation “HC1”** |  |  |
| Dexamethasone | 5 mg/m^2^/ 12 h. | Days 1 to 5 of week 5 |
| Vincristine | 1.5 mg/m^2^ | Days 1 and 6 of week 5 |
| Cytarabine | 2 g/m^2^/12 h. (2 doses) | Day 5 of week 5 |
| Methotrexate | 1 g/m^2^ (36 h. infusion) | Day 1 of week 5 |
| Cyclophosphamide | 200 mg/m^2^/12 h. (5 doses) | Days 2 to 4 of week 5 |
| PEG-asparaginase | 1,000 U/m^2^ | Day 6 of week 5 |
| TIT ^1^ |  | Day 2 (day 7) of week 5 |
| **Consolidation “HC2”** |  |  |
| Dexamethasone | 5 mg/m^2^/ 12 h. | Days 1 to 6 of week 8 |
| Cytarabine | 2 g/m^2^/12 h. (4 doses) | Days 1 and 2 of week 8 |
| Etoposide | 100 mg/m^2^/12 h. (5 doses) | Days 3 to 5 of week 8 |
| PEG-asparaginase | 1,000 U/m^2^ | Day 6 of week 8 |
| TIT ^1^ |  | Day 1 of week 8 |
| **Consolidation “HC3”** |  |  |
| Dexamethasone | 5 mg/m^2^/ 12 h. | Days 1 to 6 of week 11 |
| Vincristine | 1.5 mg/m^2^ | Days 1 and 6 of week 11 |
| Daunorubicine | 30 mg/m^2^ (24 h. infusion) | Day 5 of week 11 |
| Methotrexate | 1 g/m^2^ (36 h. infusion) | Day 1 of week 11 |
| Ifosfamide | 800 mg/m^2^/12 h. (5 doses) | Days 2 to 4 of week 11 |
| PEG-asparaginase | 1,000 U/m^2^ | Day 6 of week 11 |
| TIT ^1^ |  | Day 2 of week 11 |
| **Standard risk relapse** |  |  |
| **Induction “SIA”** |  |  |
| Dexamethasone | 10 mg/m^2^/ 12 h. | Days 1 to 5 of weeks 1 and 3 |
| Vincristine | 1.5 mg/m^2^ | Days 1 and 6 of week 1 and day 1 of week 3 |
| Methotrexate | 1 g/m^2^ (36 h. infusion) | Day 1 of week 1 |
| Cytarabine | 3 g/m^2^/12 h. | Days 1 and 2 of week 3 |
| PEG-asparaginase | 1,000 U/m^2^ | Day 4 of week 1 and 3 |
| TIT ^1^ |  | Day 1 (day 6) of week 1 and day 5 of week 3 |

**Table S2. Treatment protocol before stem cell transplantation (cont.)**

| **Consolidation “SCA1”** |  |  |
| --- | --- | --- |
| Dexamethasone | 3 mg/m^2^/ 12 h. | Days 1 to 7 of weeks 5 and 6 ^2^ |
| Vincristine | 1.5 mg/m^2^ | Day 1 of weeks 5, 6, 7 and 8 |
| Idarubicine | 6 mg/m^2^ (2 h. infusion) | Day 1 of weeks 5, 6, 7 and 8 |
| PEG-asparaginase | 1,000 U/m^2^ | Day 1 of week 5 and day 4 of week 6 |
| TIT ^1^ |  | Day 1 of weeks 5 (day 1 of week 6) and 7 |
| **Consolidation “SCA2”** |  |  |
| Cyclophosphamide | 1 g/m^2^ | Day 1 of week 9 |
| Cytarabine | 75 mg/m^2^ (15 minutes) | Days 3 to 6 of weeks 9 and 10 |
| Thioguanine | 60 mg/m^2^ | Days 1 to 7 of weeks 9 and 10 |
| TIT ^1^ |  | Day 3 of weeks 9 and 10 |
| **Consolidation “SCA3”** |  |  |
| Dexamethasone | 10 mg/m^2^/ 12 hours | Days 1 to 5 of week 13 ^2^ |
| Mercaptopurine | 100 mg/m^2^ | Days 1 to 5 of week 13 |
| Vincristine | 1.5 mg/m^2^ | Days 1 and 6 of week 13 |
| Methotrexate | 1 g/m^2^ (36 h. infusion) | Day 1 of week 13 |
| Cytarabine | 2 g/m^2^/12 h. (2 doses) | Day 5 of week 13 |
| PEG-asparaginase | 1,000 U/m^2^ | Day 6 of week 13 |
| TIT ^1^ |  | Day 1 of weeks 13 |

Abbreviations; h., hours; HC1/2/3, high risk consolidation blocks 1, 2 and 3; HIA, high risk induction “A”; SCA1/2/3, standard risk consolidation blocks 1, 2 and 3; SIA, standard risk induction “A”; TIT, triple intrathecal therapy

^1^ Age adapted doses of triple intrathecal chemotherapy: methotrexate, cytarabine and hydrocortisone 5, 16 and 10 mg, respectively for patients < 1 year old; 8, 16 and 10 mg for those ≥ 1 and < 2 year old; 10, 20 and 15 mg for those ≥ 2 and < 3 year old; 12, 30 and 20 mg for those ≥ 3 year old. Days between parentheses represent additional doses of TIT scheduled for patients with central nervous system involvement at relapse.

^2^ Dose tapering until day 2 of week 8; 5 mg/m^2^/12 h. on day 6 of week 13

**Table S3. Indications of allogeneic transplantation in patients with standard risk relapse**

|  | **Late BM isolated or combined** | | | **Early combined BM** | | | **Isolated**  **EM** | |
| --- | --- | --- | --- | --- | --- | --- | --- | --- |
|  | **MRD**  **GR ^1^** | **MRD**  **PR ^1^** | **MRD**  **ND** | **MRD**  **GR ^1^** | **MRD**  **PR ^1^** | **MRD**  **ND** | **Late** | **Early** |
| **MD** | No | Yes | Yes | Yes | Yes | Yes | No | Yes |
| **MMD** | No | Yes | No | No | Yes | Yes | No | No |

Abbreviations; BM, bone marrow; EM, extramedullary; GR, good response; MD, matched donor; MMD, mismatched donor; MRD, minimal residual disease; ND, not done or not available; PR, poor response

^1^ MRD GR is defined as < 0.1% (< 10^-3^) residual disease after re-induction by flow cytometry, and MRD PR is defined as ≥ 0.1% residual disease.

**Table S4. Patients undergoing stem cell transplantation without a scheduled indication**

| **Relapse** | **Genetics** | **Unscheduled indication for SCT ^1^** | **Donor** | **Outcome (m) ^2^** |
| --- | --- | --- | --- | --- |
| Late isolated EM (BCP) | *KMT2A*-r | Late isolated EM | MU | CCR (57) |
| Late isolated BM (BCP) | *TP53* deletion | MRD GR (< 0.01%) after re-induction | MU | CCR (5) |
| Late isolated BM (BCP) | High hyperdiploidy | MRD GR (< 0.01%) after re-induction | MR | CCR (59) |
| Late isolated BM (BCP) | B-other | MRD GR (< 0.01%) after re-induction | MU | TRD (4) |
| Late isolated BM (BCP) | B-other | MRD GR (0.06%) after re-induction | MU | CCR (53) |
| Late combined (BCP) | *IKZF1* deletion | MRD GR (0.08%) after re-induction | Haplo | TRD (8) |
| Early isolated EM (BCP) | No data | SCT from mismatched donor | Haplo | CCR (25) |

^1^ Deviations from scheduled indications for standard risk relapse (table S3)

^2^ Months since transplantation.

Abbreviations; BCP, B-cell precursor; BM, bone marrow; CCR, continuous complete remission; EM, extramedullary; GR, good response; Haplo, haploidentical; *KMT2A*-r, *KMT2A* rearrangement; m, months; MR, matched related; MRD, minimal residual disease; MU, matched unrelated; SCT, allogeneic stem cell transplantation; TRD, treatment-related death.

**Table S5. Treatment and outcome of patients failing to respond to re-induction and/or with persistent minimal residual disease**

| **Relapse** | **Disease status ^1^** | **Treatment** | **Response** | **Donor** | **Outcome (m) ^2^** |
| --- | --- | --- | --- | --- | --- |
| Early isolated BM (BCP) ^3^ | Refractory | Clofarabine, VP16, Cy | CR2 (MRD negative) | MR | CCR (41) |
| Very early isolated BM (BCP) | Refractory | Clofarabine, VP16, Cy | CR2 (MRD negative) | Haplo | TRD (2) |
| Early isolated BM (BCP) | Refractory | Clofarabine, VP16, Cy | CR2 (MRD negative) | MR | TRD (45) |
| Early isolated BM (BCP) | Refractory | TVTC | CR2 (MRD negative) | Haplo | TRD (5) |
| Late isolated BM (BCP) | Refractory | Inotuzumab | CR2 (MRD na) | Haplo | TRD (3) |
| Early isolated BM (BCP) | Persistent MRD | Blinatumomab | CR2 (MRD negative) | MU | 2^nd^ REL (7) |
| Early isolated BM (BCP) | Persistent MRD | Clofarabine, VP16, Cy | CR2 (MRD negative) | MR | CCR (78) |
| Early combined (BCP) | Persistent MRD | Clofarabine, VP16, Cy | CR2 (MRD 1.09%) | MR | 2^nd^ REL (6) |
| Late isolated BM (BCP) | Persistent MRD | Rituximab | CR2 (MRD negative) | MU | CCR (27) |
| Late isolated BM (BCP) | Persistent MRD | Blinatumomab | CR2 (MRD negative) | MU | CCR (20) |
| Late isolated BM (BCP) | Persistent MRD | Blinatumomab | CR2 (MRD negative) | Haplo | CCR (45) |
| Late combined (BCP) | Persistent MRD | Blinatumomab | CR2 (MRD negative) | Haplo | CCR (27) |
| Very early combined (T) | Persistent MRD | Daratumomab | CR2 (MRD negative) | Haplo | TRD (11) |

^1^ Disease status after re-induction and indication for salvage (rescue) treatment before SCT.

^2^ Months since transplantation.

^3^ Misclassified as standard risk relapse

Abbreviations; BCP, B-cell precursor; BM, bone marrow; CCR, continuous complete remission; CR2, second complete remission; Cy, cyclophosphamide; Haplo, haploidentical; m, months; MR, matched related; MRD, minimal residual disease; MU, matched unrelated; na, not available; REL, relapse; TRD, treatment-related death; T, T-cell immunophenotype; TVTC, topotecan, vinorelbine, thiotepa, clofarabine (reference number 1); VP16, etoposide.

**Table S6. Characteristics of B cell precursor ALL patients and donor-recipient HLA matching**

|  | **Haploidentical**  **donor** |  | **HLA-matched donor** |  |  |
| --- | --- | --- | --- | --- | --- |
|  | **n = 23** | **%** | **n = 43** | **%** | **p** |
| **Age at SCT (years) ^1^** |  |  |  |  |  |
| ≥ mean | 11 | 48 | 24 | 56 | 0.5355 |
| < mean | 12 | 52 | 19 | 44 |  |
| **Time to relapse ^2^** |  |  |  |  |  |
| Very early | 4 | 17 | 5 | 11.6 | 0.8267 |
| Early | 11 | 48 | 21 | 48.8 |  |
| Late | 8 | 35 | 17 | 39.6 |  |
| **Site of relapse** |  |  |  |  |  |
| Isolated BM | 17 | 74 | 28 | 65.1 | 0.7654 |
| Combined BM | 5 | 22 | 11 | 25.6 |  |
| Isolated EM | 1 | 4 | 4 | 9.3 |  |
| **Risk-group at relapse** |  |  |  |  |  |
| High | 14 | 61 | 17 | 39.5 | 0.1637 |
| Standard | 9 | 39 | 26 | 60.5 |  |
| **MRD before SCT ^3^** |  |  |  |  |  |
| ≥ 0.01% | 1 | 4.5 | 7 | 17 | 0.1637 |
| < 0.01% | 21 | 95.5 | 35 | 83 |  |
| No data | 1 |  | 1 |  |  |
| **HLA matching (matched alleles) ^4^** |  |  |  |  |  |
| 9/10 | na | na | 8 | 18.6 | na |
| 10/10 | na | na | 29 ^5^ | 67.4 | na |
| Umbilical cord blood (5 to 6/6) | na | na | 6 | 14 | na |
| No data | na | na | 0 | 0 | na |

Abbreviations; ALL, acute lymphoblastic leukemia; BM, bone marrow; EM extramedullary; HLA, human leukocyte antigen; MRD, minimal residual disease; na, not applicable; SCT, allogeneic stem cell transplantation;

^1^ Mean age 8 years (range: 1 to 19)

^2^ Very early relapse: < 6 months after the end of first-line treatment and < 18 months after primary diagnosis. Early relapse: < 6 months after the end of first-line treatment but ≥ 18 months after primary diagnosis. Late relapse: ≥ 6 months after the end of first-line treatment.

^3^ Five patients had MRD ≥ 0.1% (4 in the haploidentical and 1 in the HLA compatible donor group)

^4^ Patients without available data were excluded from the analysis of the corresponding variable.

^5^ Thirteen related and 16 unrelated donors.

**Table S7. Transplantation characteristics of B-cell precursor ALL patients**

|  | **Haploidentical**  **donor** |  | **HLA-matched donor** |  |  |
| --- | --- | --- | --- | --- | --- |
|  | **n = 23** | **%** | **n = 43** | **%** | **p** |
| **Conditioning** |  |  |  |  |  |
| TT + Bu + Flu | 14 | 61 | 16 | 37 | 0.0659 |
| Bu + Cy ± TT | 0 | 0 | 4 | 9 | 0.1313 |
| TBI + VP16 | 1 | 4 | 5 | 12 | 0.3269 |
| TBI + Cy | 0 | 0 | 7 | 16 | **0.0407** |
| TLI + TT + Flu + L-PAM | 5 | 22 | 2 | 5 | **0.0317** |
| TBI + TT + Cy | 0 | 0 | 4 | 9 | 0.1313 |
| Other ^1^ | 3 | 13 | 5 | 12 | 0.8667 |
| **TBI-based conditioning** |  |  |  |  |  |
| Yes | 3 | 13 | 20 | 46.5 | **0.0065** |
| No | 20 | 87 | 23 | 53.5 |  |
| **Stem cell source** |  |  |  |  |  |
| Peripheral blood | 18 | 78.3 | 20 | 46.5 | **0.027** |
| Bone marrow | 5 | 21.7 | 17 | 39.5 |  |
| Umbilical cord blood | 0 |  | 6 | 14 |  |
| ***Ex vivo* graft manipulation ^2, 3^** |  |  |  |  |  |
| No manipulation | 9 | 40.9 | 39 | 90.7 | **<0.0001** |
| αβ T-cell and CD19+ depletion | 8 | 36.4 | 0 | 0 | **<0.0001** |
| CD45RA+ depletion | 4 | 18.2 | 4 | 9.3 | 0.3025 |
| CD3+ and CD19+ depletion | 1 | 4.5 | 0 | 0 | 0.1589 |
| No data | 1 |  | 0 |  |  |
| **CD34+ cell dose infused ^2, 4^** |  |  |  |  |  |
| ≥ mean | 11 | 50 | 19 | 50 | 1 |
| < mean | 11 | 50 | 19 | 50 |  |
| ≥ 2 x 10^6^ CD34+/kg | 21 | 81.8 | 34 (29 ^5^) | 89.5 (90.6 ^5^) | 0.41 (0.5) |
| ≥ 5 x 10^6^ CD34+/kg | 13 | 50 | 21 (18 ^5^) | 55.3 (56.2 ^5^) | 0.77 (0.83) |
| No data | 1 |  | 5 |  |  |
| **GVHD prophylaxis ^2^** |  |  |  |  |  |
| Cyclosporine + methotrexate | 0 | 0 | 22 | 51.2 | **<0.0001** |
| Cyclosporine | 8 | 36.4 | 6 | 14 | **0.0376** |
| Cyclosporine + MF | 5 | 22.7 | 1 | 2.3 | **0.0072** |
| Tacrolimus + methotrexate | 0 | 0 | 4 | 9.3 | 0.1398 |
| Tacrolimus + MF | 3 | 13.6 | 0 | 0 | **0.0132** |
| Tacrolimus | 1 | 4.5 | 3 | 7 | 0.6995 |
| MF | 1 | 4.5 | 2 | 4.6 | 0.9847 |
| Cyclosporine + prednisolone | 0 | 0 | 1 | 2.3 | 0.471 |
| None ^6^ | 4 | 18.2 | 4 | 9.3 | 0.3025 |
| No data | 1 |  | 0 |  |  |
| **Serotherapy ^2^** |  |  |  |  |  |
| Yes | 3 | 13.6 | 26 | 60.5 | **0.0003** |
| No | 19 | 86.4 | 17 | 39.5 |  |
| No data | 1 |  | 0 |  |  |

Abbreviations; ALL, acute lymphoblastic leukemia; Bu, busulphan; Cy, cyclophosphamide; Flu, fludarabine; GVHD, graft versus host disease; L-PAM, melphalan; na, not applicable; MF, mycophenolate mofetil; TBI, total body irradiation; TLI, total lymphoid irradiation; irradiation; TT, Thiotepa; VP16, etoposide

^1^ Other conditioning regimens: Bu + TT; Bu + Flu; Cy + VP16; TBI + Flu ± Cy or TT; TBI + Cy + VP16; TBI + TT.

^2^ Patients without available data were excluded from the analysis of the corresponding variable.

^3^ Patients without graft manipulation (T-cell replete grafts) in the haploidentical donor group received post-transplant cyclophosphamide.

^4^ Mean CD34+ cell dose infused was 5.07 x 10^6^/kg (range 0.35-14.6).

^5^ Excluding 6 patients who received umbilical cord blood transplantation (n = 32)

^6^ One and three patients in the haploidentical donor group received no pharmacologic GVHD prophylaxis after ºαβ T-cell depletion and CD45RA+, respectively; 4 in the HLA compatible donor group received no pharmacologic GVHD prophylaxis after CD45RA+ graft depletion.

**Table S8. CD34+ cell dose in patients with primary or secondary graft failure**

| **Donor** | **Graft manipulation** | **Stem cell source** | **CD34+ cell dose**  **(x 10^6^/kg)** | **Graft failure** |
| --- | --- | --- | --- | --- |
| Matched related | No | Bone marrow | 1.74 | Primary |
| Matched unrelated | CD45RA depletion | Peripheral blood | 7.34 | Primary |
| Haploidentical | CD45RA depletion | Peripheral blood | 5.83 | Secondary |
| Haploidentical | αβ depletion | Peripheral blood | 4.1 | Primary |
| Haploidentical | No data | Peripheral blood | No data | Secondary |
| Haploidentical | αβ depletion | Peripheral blood | 10.26 | Primary |

**Table S9. Early complications after stem cell transplantation and chronic GVHD of B cell precursor ALL patients**

|  | **Haploidentical**  **donor** |  | **HLA-matched donor** |  |  |
| --- | --- | --- | --- | --- | --- |
|  | **n = 23** | **%** | **n = 43** | **%** | **p** |
| **Graft failure ^1^** |  |  |  |  |  |
| Yes | 4 | 17.4 | 1 | 2.4 | **0.0299** |
| No | 19 | 82.6 | 41 | 97.6 |  |
| No data | 0 |  | 1 |  |  |
| **aGVHD** |  |  |  |  |  |
| Grade 1 | 3 | 13 | 3 | 7 | 0.7433 |
| Grade ≥ 2 | 7 | 30.5 | 15 | 34.9 |  |
| No | 13 | 56.5 | 25 | 58.1 |  |
| **Grade ≥ 3 infections** |  |  |  |  |  |
| Yes | 14 | 60.9 | 21 | 48.8 | 0.3507 |
| No | 9 | 39.1 | 22 | 51.2 |  |
| **SOS (any grade)** |  |  |  |  |  |
| Yes | 2 | 8.7 | 8 | 18.6 | 0.2847 |
| No | 21 | 91.3 | 35 | 81.4 |  |
| **Other CTCAE grade ≥ 3 ^1^** |  |  |  |  |  |
| Yes | 10 | 45.4 | 15 | 37.5 | 0.5413 |
| No | 12 | 54.6 | 25 | 62.5 |  |
| No data | 1 |  | 3 |  |  |
| **cGVHD ^1, 2^** |  |  |  |  |  |
| Yes | 1 | 5.3 | 4 | 11.4 | 0.4554 |
| No | 18 | 94.7 | 31 | 88.6 |  |
| No data | 1 |  | 1 |  |  |

Abbreviations; aGVHD, acute graft versus host disease; cGVHD, chronic graft versus host disease; CTCAE, Common Terminology Criteria for Adverse Events version 3.0; SOS, sinusoidal obstructive syndrome (any grade)

^1^ Patients without available data were excluded from the analysis of the corresponding variable.

^2^ Proportions of patients with any grade cGVHD among those surviving > 100 days after transplantation (20 in the haploidentical and 36 in the HLA-matched donor group)

**Table S10. Overall results of B-cell precursor ALL patients: probability (%) and 95% CI**

|  | **Haploidentical**  **donor** | **HLA-matched**  **donor** | **p** |
| --- | --- | --- | --- |
| **OS** | 59.4% (42-84.1) | 63.9% (50.8-80.5) | 0.9 |
| **LFS** | 52.2% (35.3-77.2) | 38.4% (26.2-56.4) | 0.6 |
| **EFS** | 43.5% (27.3-69.3) | 36.1% (24.1-54) | 0.9 |
| **CIR** | 26.1% (10.3-45.3) | 40.5% (25.5-55.1) | 0.63 |
| **TRM** | 21.7% (7.6-40.5) | 21% (10.3-34.3) | 0.87 |
| **Cumulative incidence of cGVHD** | 5% (0.3-21) | 14.8% (4.5-30.7) | 0.43 |
| **GLFS** | 47.8% (31.2-73.3) | 31.2% (19.9-49) | 0.5 |

Abbreviations; ALL, acute lymphoblastic leukemia; cGVHD, chronic graft versus host disease; GLFS, cGVHD-free and leukemia-free survival; CIR, cumulative incidence of relapse; EFS, event free survival; LFS, leukemia free survival; OS, overall survival; TRM, treatment related mortality.

**Table S11. Factors influencing leukemia-free survival in B cell precursor ALL patients: univariate analysis**

|  | **N. of patients** | **Events** | **Probability (%)** | **95% CI** | ***P* value** |
| --- | --- | --- | --- | --- | --- |
| **Risk group at relapse** |  |  |  |  |  |
| Standard risk | 35 | 17 | 50.5 | 36.2-70.4 | 0.1 |
| High risk | 31 | 20 | 31.9 | 21.5-56.8 |  |
| **MRD before SCT** |  |  |  |  |  |
| < 0.01% | 56 | 26 | 52.1 | 40.4-67.3 | **0.006** |
| ≥ 0.01% | 8 | 9 | 0 |  |  |
| No data | 2 |  |  |  |  |
| **Conditioning regimen** |  |  |  |  |  |
| TBI-based | 23 | 10 | 58.8 | 37.5-80.1 | 0.1 |
| Chemotherapy-based | 43 | 27 | 36.8 | 24.8-54.6 |  |
| No data | 0 |  |  |  |  |
| **CD34+ cell dose infused** |  |  |  |  |  |
| < mean | 30 | 20 | 49.8 | 35.1-70.6 | 0.8 |
| ≥ mean | 30 | 16 | 41.3 | 26.3-64.7 |  |
| No data | 6 |  |  |  |  |
| **Grade 1/2 aGVHD** |  |  |  |  |  |
| Yes | 12 | 5 | 549 | 30.8-94.6 | 0.5 |
| No | 54 | 51 | 40.7 | 29.5-56.2 |  |
| **Any grade cGVHD** |  |  |  |  |  |
| Yes | 5 | 1 | 80 | 51.6-100 | 0.06 |
| No | 61 | 36 | 40 | 29.3-64.6 |  |

Abbreviations; aGVHD, acute graft versus host disease; ALL, acute lymphoblastic leukemia; cGVHD, chronic graft versus host disease; MRD, minimal residual disease; N., numbers; SCT, stem cell transplantation; TBI, total body irradiation.

**Table S12. Factors influencing leukemia-free survival in B cell precursor ALL patients: multivariate analysis**

|  | **Hazard ratio (95%CI)** | **p value** |
| --- | --- | --- |
| **MRD before SCT ≥ 0.01%** | 2.81 (1.3056-6.088) | 0.00836 |

Abbreviations; ALL, acute lymphoblastic leukemia; cGVHD, chronic graft versus host disease; MRD, minimal residual disease; SCT, stem cell transplantation; TBI, total body irradiation.

**Table S13. Reported results after SCT in pediatric patients with hematological malignancies undergoing allogeneic stem cell transplantation (estimated rates presented as percentages)**

| **Study / study group** | **Year of publication** | **OS** | **LFS/**  **PFS** | **EFS** | **CIR** | **TRM/**  **NRM** | **cGVHD** | **GLFS** | **FU (y)** | **Ref.** |
| --- | --- | --- | --- | --- | --- | --- | --- | --- | --- | --- |
| EBMT Registry ^1^ | 2010 | 39 | 34 | - | 36 | 30 | - | - | 5 | 2 |
| Peking University ^2^ | 2013 | 60.2 | 56.6 | - | 34.3 | 19.5 | 40.1 | - | 5 | 3 |
| BFM ^3^ | 2015 | 73-79 | - | 67-71 | 22-24 | 3-10 | - | - | 4 | 4 |
| AIEOP-GITMO ^4^ | 2016 | 72 | 61 | - | 24 | 9 | 0-7 | - | 1 | 5 |
| China ^5^ | 2016 | 82 | 71 | - | 16.1 | 12.8 | 64 | - | 2 | 6 |
| China ^6^ | 2016 | 69.6 | 57.2 | - | 24.1 | 18.8 | 6.3 | - | 2 | 6 |
| Madrid (Spain) ^7^ | 2016 | - | 52 | - | 32 | 23 | 46 | - | 2 | 7 |
| Montevideo ^4^ | 2016 | 48 | - | - | 23 | 26 | 53 | - | - | 8 |
| Montevideo ^7^ | 2016 | 47 | - | - | 31 | 24 | 9 | - | - | 8 |
| Monterrey ^4^ | 2016 | 50 | - | 33 | 40 | 36 | - | - | 1 | 9 |
| NCT0181010 ^8^ | 2017 | 72 | 71 | 69.5 | 24 | 5 | 5 | 71 | 5 | 10 |
| BFM ^9^ | 2018 | 56 | - | 52 | 31 | 19 | 15 | - | 4 | 11 |
| Texas Children’s Hospital ^10^ | 2018 | 40 |  | 35 | 47 | 15 | - | - | 3 | 12 |
| JSHCT ^11^ | 2019 | 75.8 |  | 69.3 | 20.1 | 9.1 | 29.5 | - | 3 | 13 |
| Multinational ^12^ | 2019 | 63 | - | 57 | 30 | 14 | 21 | - | 4 | 14 |
| BFM ALL SCT 2007 ^3^ | 2019 | 68-72 | - | 61-65 | 24-25 | 10-14 | 25-37 | - | 4 | 15 |
| EBMT ^13^ (TBI) | 2020 | 58.8 | 53.7 | - | 30.6 | 15.7 | 21.2 | - | 5 | 16 |
| EBMT ^13^ (CC) | 2020 | 35.9 | 29.4 | - | 49.3 | 21.3 | 26 | - | 5 | 16 |
| SJCRH ^14^ | 2020 | 88.1 | - | 77.7 | 11.5 | 5.6  (1 y) | 25.9 | - | 3 | 17 |
| Arizona ^4^ | 2020 | 84 | 74.3 | - | 17.6 | 9.5 | 18.1 | - | 2 | 18 |
| NCT01949129 ^3^ (TBI) | 2021 | 91 | - | 75 | 22 | 4 | - | 72 | 2 | 19 |
| NCT01949129 ^3^ (CC) | 2021 | 74 | - | 46 | 44 | 9 | - | 51 | 2 | 19 |
| ALLR3 & ALL-REZ BFM 2002 ^15^ | 2021 | 52.7 | 46.4 | - | 31.6 | 14.9 | - | - | - | 20 |
| ALL-SCT-(I)BFM 2013 & 2007 ^12^ | 2021 | 65 | - | 53 | 36 | 9 | 11-17 | - | 4 | 21 |
| HIUNJ ^8^ | 2022 | 58 | 45 | - | 34 | 21 | 23 | - | - | 22 |
| HIUNJ ^16^ | 2022 | 53 | 45 |  | 32 | 23 | 32 | - | - | 22 |

Abbreviations; ALL, acute lymphoblastic leukemia; AML, acute myeloblastic leukemia; BFM, Berlin-Frankfurt, Muenster; CC, chemotherapy-based conditioning; cGVHS, cumulative incidence of chronic graft versus host disease; CIR, cumulative incidence of relapse; CR1, first complete remission; CR2 second, complete remission; EBMT, European Bone Marrow Transplantation; EFS, event free survival; FU, follow up; GLFS, graft versus host disease and leukemia free survival; HIUNJ, Hospital Infantil Universitario Niño Jesús; JSHCT, Japan Society for Hematopoietic Cell Transplantation; LFS, leukemia free survival; MMD, mismatched donor; MRD, matched related donor; MUR, matched unrelated donor; NRM, nonrelapse mortality; OS, overall survival; PFS, progression free survival; Ref., reference; SCT, allogenic stem cell transplantation; SJCRH, Saint Jude Children`s Research Hospital; TBI, total body irradiation; TRM, treatment related mortality; y, years

^1^ Data from patients with ALL who received haploidentical transplantation in CR2

^2^ OS, LFS, CIR and TRM data from 59 patients with ALL in CR2 undergoing unmanipulated haploidentical transplantation without post-transplant high-dose cyclophosphamide (“Beijing protocol”), cGVHD data from 193 patients with ALL and other hematological malignancies in different remission status

^3^ Include patients with ALL in CR1 and ≥ CR2 undergoing SCT from MRD and MUD

^4^ Include patients with ALL, AML and other hematological malignancies in CR1 and ≥ CR2 undergoing haploidentical transplantation with post-transplant high-dose cyclophosphamide

^5^ Data from patients with ALL, including CR1, CR2 and ≥ CR3, undergoing unmanipulated haploidentical transplantation without post-transplant high-dose cyclophosphamide (“Beijing protocol”)

^6^ Data from patients with ALL, including CR1, CR2 and ≥ CR3, undergoing umbilical cord blood transplantation

^7^ Include patients with ALL, AML and other hematological malignancies in CR1 and ≥ CR2 undergoing haploidentical transplantation using CD3/CD19-depleted grafts

^8^ Include patients with ALL and AML in CR1 and ≥ CR2 undergoing haploidentical transplantation after αβ T-cell depletion

^9^ Data from patients with ALL in CR1 and ≥ CR2 undergoing transplantation from MMD (CIR of patients undergoing SCT in CR2).

^10^ Data from patients with ALL in different remission status (including CR1) undergoing haploidentical transplantation using CD34+ selected grafts

^11^ Data from patients with ALL and AML in CR1 and CR2 undergoing SCT from MRD, MUD and MMD

^12^ Data from patients with ALL in CR1 and ≥ CR2 undergoing SCT from MRD, MUD and MMD

^13^ Data from patients with ALL in CR2 undergoing SCT from MRD and MUD after TBI-based and CC

^14^ Include patients with ALL, AML and other hematological malignancies in CR1 and ≥ CR2 undergoing haploidentical transplantation using CD45RA-depleted grafts

^15^ Data from patients with ALL in CR2 undergoing SCT from MRD, MUD and MMD

^16^ Include patients with ALL and AML in different remission status undergoing haploidentical transplantation using CD3/CD19-depleted grafts


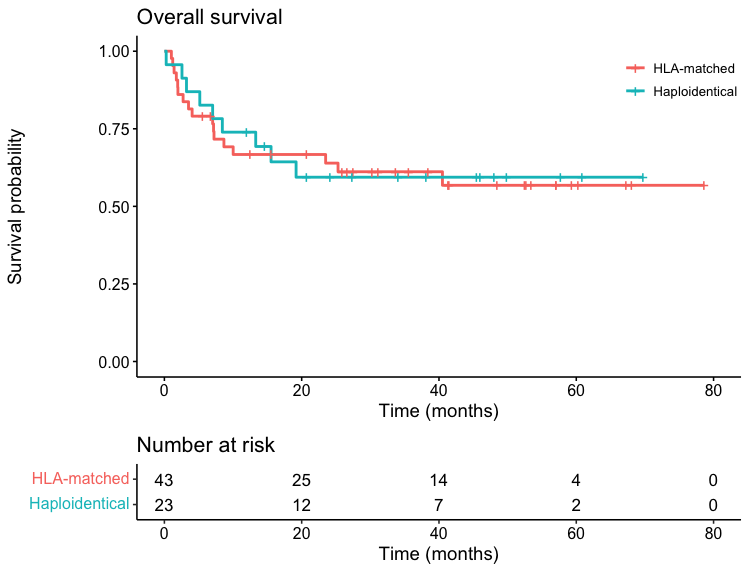

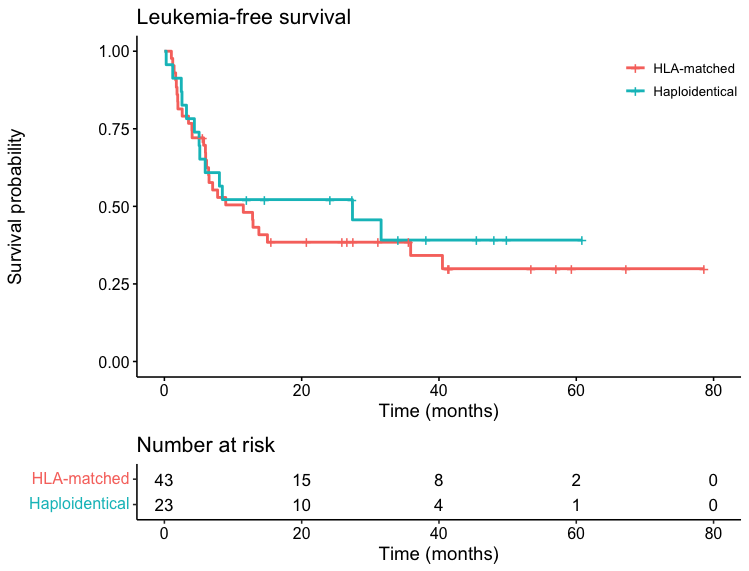

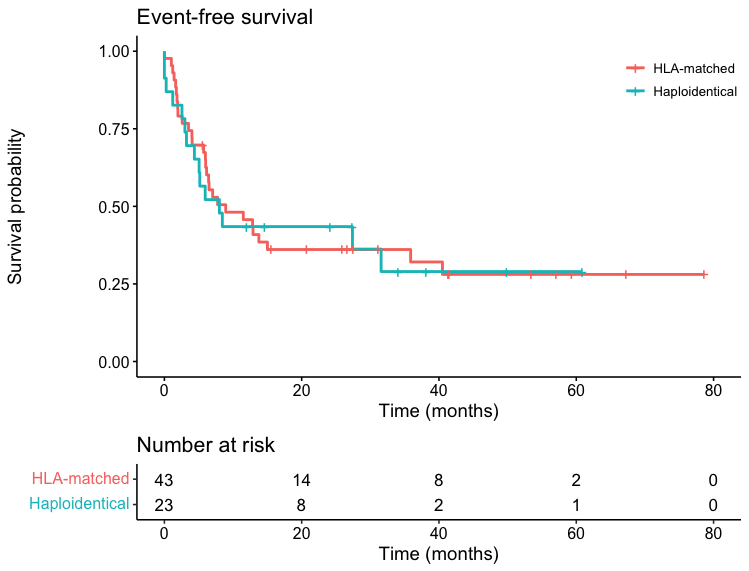

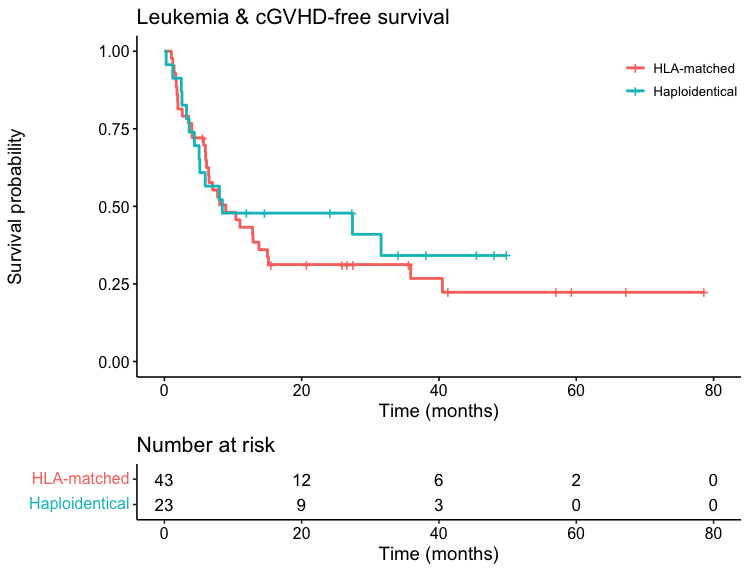


**Supplementary Figure S1.** Two year overall survival (59.4% *versus* 63.9%; p = 0.9), leukemia free survival (52.2% *versus* 38.4%; p = 0.6), event-free survival (43.5% *versus* 36.1%; p = 0.9), and chronic graft versus host disease free and leukemia free survival (47.8% versus 31.2%; p = 0.5) among patients with B-cell precursor acute lymphoblastic leukemia undergoing transplantation from haploidentical (blue lines) and HLA-matched (red lines) donors.

**Supplementary Figure S2.** Two year cumulative incidence of second relapse (26.1% *versus* 40.5%; p = 0.63), treatment related mortality (21.7% *versus* 21%; p = 0.87), and chronic graft versus host disease (5% versus 14.8%; p = 0.43) among patients with B-cell precursor acute lymphoblastic leukemia undergoing transplantation from haploidentical (dashed lines) and HLA-matched donors (solid lines).


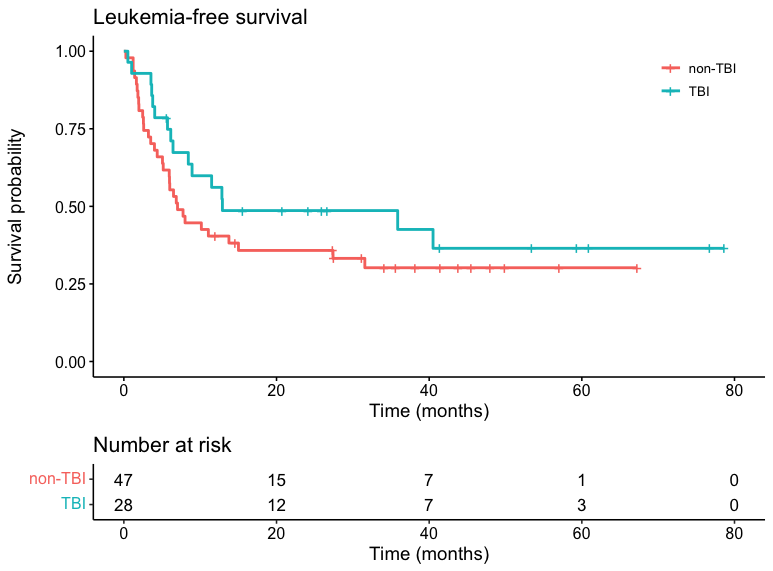


**Supplementary Figure S3.** Two year leukemia free survival (48.6% *versus* 35.8%; p = 0.2), among patients undergoing transplantation after TBI-conditioning (blue lines) and non-TBI conditioning (red lines) regimens.

References

1. Shukla N, Kobos R, Renaud T, Steinherz LJ, Steinherz PG. [Phase II trial of clofarabine with topotecan, vinorelbine, and thiotepa in pediatric patients with relapsed or refractory acute leukemia.](https://pubmed.ncbi.nlm.nih.gov/24115731/) Pediatr Blood Cancer. 2014 Mar;61(3):431-5. doi: 10.1002/pbc.24789. Epub 2013 Sep 24.
2. [Klingebiel](https://pubmed.ncbi.nlm.nih.gov/?term=Klingebiel+T&cauthor_id=20040760) T, [Cornish](https://pubmed.ncbi.nlm.nih.gov/?term=Cornish+J&cauthor_id=20040760) J, [Labopin](https://pubmed.ncbi.nlm.nih.gov/?term=Labopin+M&cauthor_id=20040760) M, [Locatelli](https://pubmed.ncbi.nlm.nih.gov/?term=Locatelli+F&cauthor_id=20040760) F, [Darbyshire](https://pubmed.ncbi.nlm.nih.gov/?term=Darbyshire+P&cauthor_id=20040760) P, [Handgretinger](https://pubmed.ncbi.nlm.nih.gov/?term=Handgretinger+R&cauthor_id=20040760) R, et al. Results and factors influencing outcome after fully haploidentical hematopoietic stem cell transplantation in children with very high-risk acute lymphoblastic leukemia: impact of center size: an analysis on behalf of the Acute Leukemia and Pediatric Disease Working Parties of the European Blood and Marrow Transplant group. Blood. 2010;115(17):3437-46.
3. Liu DH, Xu LP, Liu KY, Wang Y, Chen H, Han W, et al. Long term outcomes of unmanipulated haploidentical HSCT for paediatric patients with acute leukaemia. Bone Marrow Transpl. 2013;48:1519–24.
4. Peters C, Schrappe M, von Stackelberg A, Schrauder A, Bader P, Ebell W, et al. [Stem-cell transplantation in children with acute lymphoblastic leukemia: A prospective international multicenter trial comparing sibling donors with matched unrelated donors-The ALL-SCT-BFM-2003 trial.](https://pubmed.ncbi.nlm.nih.gov/25753432/) J Clin Oncol 2015;33(11):1265-74.
5. Berger M, Lanino E, Cesaro S, Zecca M, Vassallo E, Faraci M, et al. Feasibility and outcome of haploidentical hematopoietic stem cell transplantation with post-transplant high-dose cyclophosphamide for children and adolescents with hematologic malignancies: an AIEOP-GITMO Retrospective Multicenter Study. Biol Blood Marrow Transplant. 2016;22(5):902-909.
6. [Mo](https://pubmed.ncbi.nlm.nih.gov/?term=Mo+XD&cauthor_id=27356906) X-D, [Tang](https://pubmed.ncbi.nlm.nih.gov/?term=Tang+BL&cauthor_id=27356906) B-L, [Zhang](https://pubmed.ncbi.nlm.nih.gov/?term=Zhang+XH&cauthor_id=27356906) X-H, [Zheng](https://pubmed.ncbi.nlm.nih.gov/?term=Zheng+CC&cauthor_id=27356906) C-C,  [Xu](https://pubmed.ncbi.nlm.nih.gov/?term=Xu+LP&cauthor_id=27356906) L-P, [Zhu](https://pubmed.ncbi.nlm.nih.gov/?term=Zhu+XY&cauthor_id=27356906) X-Y, et al. Comparison of outcomes after umbilical cord blood and unmanipulated haploidentical hematopoietic stem cell transplantation in children with high-risk acute lymphoblastic leukemia. Int J Cancer 2016;139(9):2106-15.
7. Diaz MA, Perez-Martinez A, Herrero B, Deltoro N, Martinez I, Ramírez M, et al. Prognostic Factors and Outcomes for Pediatric Patients Receiving an Haploidentical Related Allogeneic Transplantation Using CD3/CD19-Depleted Grafts. Bone Marrow Transplant 2016;51:1211–6.
8. Dufort G, Castillo L, Pisano S, Castiglioni M, Carolina P, Andrea I, et al. Haploidentical hematopoietic stem cell transplantation in children with high-risk hematologic malignancies: outcomes with two different strategies for GvHD prevention. Ex vivo T-cell depletion and post-transplant cyclophosphamide: 10 years of experience at a single center. Bone Marrow Transpl. 2016;51:1354–60.
9. Gonzalez-Llano O, Gonzalez-Lopez EE, Ramirez-Cazares AC, Marcos-Ramirez ER, Ruiz-Arguelles GJ, Gomez-Almaguer D. Haploidentical peripheral blood stem cell transplantation with posttransplant cyclophosphamide in children and adolescents with hematological malignancies. Pediatr Blood Cancer. 2016;63:2033–7.
10. Locatelli F, Merli P, Pagliara D, Li Pira G, Falco M, Pende D, et al. Outcome of Children With Acute Leukemia Given HLA-Haploidentical HSCT After αβ T-Cell and B-Cell Depletion. Blood 2017;130(5):677–68.
11. Dalle JH, Balduzzi A, Bader P, Lankester A, Yaniv I, Wachowiak J, et al. [Allogeneic Stem Cell Transplantation from HLA-Mismatched Donors for Pediatric Patients with Acute Lymphoblastic Leukemia Treated According to the 2003 BFM and 2007 International BFM Studies: Impact of Disease Risk on Outcomes.](https://pubmed.ncbi.nlm.nih.gov/29772352/) Biol Blood Marrow Transplant. 2018 Sep;24(9):1848-1855.
12. Yanir AD, Martinez CA, Sasa G, Leung K, Gottschalk S, Omer B, et al. Current Allogeneic Hematopoietic Stem Cell Transplantation for Pediatric Acute Lymphocytic Leukemia: Success, Failure and Future Perspectives-A Single-Center Experience, 2008 to 2016. Biol Blood Marrow Transplant. 2018;24(7):1424-1431.
13. [Kato](https://pubmed.ncbi.nlm.nih.gov/?term=Kato+M&cauthor_id=29795428) M, [Kurata](https://pubmed.ncbi.nlm.nih.gov/?term=Kurata+M&cauthor_id=29795428) M, [Kanda](https://pubmed.ncbi.nlm.nih.gov/?term=Kanda+J&cauthor_id=29795428) J, [Kato](https://pubmed.ncbi.nlm.nih.gov/?term=Kato+K&cauthor_id=29795428) K, [Tomizawa](https://pubmed.ncbi.nlm.nih.gov/?term=Tomizawa+D&cauthor_id=29795428) D, [Kudo](https://pubmed.ncbi.nlm.nih.gov/?term=Kudo+K&cauthor_id=29795428) K, et al. Impact of graft-versus-host disease on relapse and survival after allogeneic stem cell transplantation for pediatric leukemia. Bone Marrow Transplant. 2019;54(1):68-75.
14. [Bader](https://pubmed.ncbi.nlm.nih.gov/?term=Bader+P&cauthor_id=31714961) P, [Salzmann-Manrique](https://pubmed.ncbi.nlm.nih.gov/?term=Salzmann-Manrique+E&cauthor_id=31714961) E, [Balduzzi](https://pubmed.ncbi.nlm.nih.gov/?term=Balduzzi+A&cauthor_id=31714961) A, [Dalle](https://pubmed.ncbi.nlm.nih.gov/?term=Dalle+JH&cauthor_id=31714961) J-H, [Woolfrey](https://pubmed.ncbi.nlm.nih.gov/?term=Woolfrey+AE&cauthor_id=31714961) AE, [Bar](https://pubmed.ncbi.nlm.nih.gov/?term=Bar+M&cauthor_id=31714961) M, et al. More precisely defining risk peri-HCT in pediatric ALL: pre- vs post-MRD measures, serial positivity, and risk modeling. Blood Adv 2019;3(21):3393-3405.
15. Balduzzi A, Dalle JH, Wachowiak J, Yaniv I, Yesilipek A, Sedlacek P, et al.  [Transplantation in Children and Adolescents with Acute Lymphoblastic Leukemia from a Matched Donor versus an HLA-Identical Sibling: Is the Outcome Comparable? Results from the International BFM ALL SCT 2007 Study.](https://pubmed.ncbi.nlm.nih.gov/31319153/) Biol Blood Marrow Transplant 2019;25(11):2197-2210
16. Willasch AM, Peters C, Sedláček P, Dalle JH, Kitra-Roussou V, Yesilipek A, et al. [Myeloablative conditioning for allo-HSCT in pediatric ALL: FTBI or chemotherapy?-A multicenter EBMT-PDWP study.](https://pubmed.ncbi.nlm.nih.gov/32203263/) Bone Marrow Transplant 2020;55(8):1540-1551.
17. Mamcarz E, Madden R, Qudeimat A, Srinivasan A, Talleur A, Sharma A, et al. Improved survival rate in T-cell depleted haploidentical hematopoietic cell transplantation over the last 15 years at a single institution. Bone Marrow Transpl. 2020;55:929–38.
18. Katsanis E, Sapp LN, Reid SC, Reddivalla N, Stea B. T-cell replete myeloablative haploidentical bone marrow transplantation is an effective option for pediatric and young adult patients with high-risk hematologic malignancies. Front Pediatr. 2020;8:282.
19. Peters C, Dalle JH, Locatelli F, Poetschger U, Sedlacek P, Buechner J, et al. [Total Body Irradiation or Chemotherapy Conditioning in Childhood ALL: A Multinational, Randomized, Noninferiority Phase III Study.](https://pubmed.ncbi.nlm.nih.gov/33332189/) J Clin Oncol 2021;39(4):295-307.
20. [Eckert](https://pubmed.ncbi.nlm.nih.gov/?term=Eckert+C&cauthor_id=34010787) C, [Parker](https://pubmed.ncbi.nlm.nih.gov/?term=Parker+C&cauthor_id=34010787) C, [Moorman](https://pubmed.ncbi.nlm.nih.gov/?term=Moorman+AV&cauthor_id=34010787) AV, [Irving](https://pubmed.ncbi.nlm.nih.gov/?term=Irving+JA&cauthor_id=34010787) JA, [Kirschner-Schwabe](https://pubmed.ncbi.nlm.nih.gov/?term=Kirschner-Schwabe+R&cauthor_id=34010787) R, [Stefanie Groeneveld-Krentz](https://pubmed.ncbi.nlm.nih.gov/?term=Groeneveld-Krentz+S&cauthor_id=34010787) S, et al. Risk factors and outcomes in children with high-risk B-cell precursor and T-cell relapsed acute lymphoblastic leukaemia: combined analysis of ALLR3 and ALL-REZ BFM 2002 clinical trials. Eur J Cancer 2021;151:175-189.
21. Dalle JH, Balduzzi A, Bader P, Pieczonka A, Yaniv I, Lankester A, et al. [The impact of donor type on the outcome of pediatric patients with very high risk acute lymphoblastic leukemia. A study of the ALL SCT 2003 BFM-SG and 2007-BFM-International SG.](https://pubmed.ncbi.nlm.nih.gov/32753706/) Bone Marrow Transplant. 2021 Jan;56(1):257-266.
22. Gonzalez-Vicent M, Molina B, Lopez I, Zubicaray J, Ruiz J, Vicario JL, et al. T-Cell Depleted Haploidentical Transplantation in Children With Hematological Malignancies: A Comparison Between CD3+/CD19+ and TCRab+/CD19+ Depletion Platforms. Front. Oncol 2022. 12:884397.
